# Gene knockout inference with variational graph autoencoder learning single-cell gene regulatory networks

**DOI:** 10.1093/nar/gkad450

**Published:** 2023-05-29

**Authors:** Yongjian Yang, Guanxun Li, Yan Zhong, Qian Xu, Bo-Jia Chen, Yu-Te Lin, Robert S Chapkin, James J Cai

**Affiliations:** Department of Electrical and Computer Engineering, Texas A&M University, College Station, TX 77843, USA; Department of Statistics, Texas A&M University, College Station, TX 77843, USA; Key Laboratory of Advanced Theory and Application in Statistics and Data Science-MOE, School of Statistics, East China Normal University, 3663 North Zhongshan Road, Shanghai 200062, China; Department of Veterinary Integrative Biosciences, Texas A&M University, College Station, TX 77843, USA; Graduate Institute of Microbiology and Public Health, College of Veterinary Medicine, National Chung Hsing University, Taichung 402, Taiwan; Graduate Institute of Biomedical Electronics and Bioinformatics, National Taiwan University, Taipei, Taiwan; Program in Integrative & Complex Diseases, Department of Nutrition, Texas A&M University, College Station, TX 77843, USA; Department of Electrical and Computer Engineering, Texas A&M University, College Station, TX 77843, USA; Department of Veterinary Integrative Biosciences, Texas A&M University, College Station, TX 77843, USA; Interdisciplinary Program of Genetics, Texas A&M University, College Station, TX 77843, USA

## Abstract

In this paper, we introduce Gene Knockout Inference (GenKI), a virtual knockout (KO) tool for gene function prediction using single-cell RNA sequencing (scRNA-seq) data in the absence of KO samples when only wild-type (WT) samples are available. Without using any information from real KO samples, GenKI is designed to capture shifting patterns in gene regulation caused by the KO perturbation in an unsupervised manner and provide a robust and scalable framework for gene function studies. To achieve this goal, GenKI adapts a variational graph autoencoder (VGAE) model to learn latent representations of genes and interactions between genes from the input WT scRNA-seq data and a derived single-cell gene regulatory network (scGRN). The virtual KO data is then generated by computationally removing all edges of the KO gene—the gene to be knocked out for functional study—from the scGRN. The differences between WT and virtual KO data are discerned by using their corresponding latent parameters derived from the trained VGAE model. Our simulations show that GenKI accurately approximates the perturbation profiles upon gene KO and outperforms the state-of-the-art under a series of evaluation conditions. Using publicly available scRNA-seq data sets, we demonstrate that GenKI recapitulates discoveries of real-animal KO experiments and accurately predicts cell type-specific functions of KO genes. Thus, GenKI provides an in-silico alternative to KO experiments that may partially replace the need for genetically modified animals or other genetically perturbed systems.

## Graphical Abstract

**Graphical Abstract:**
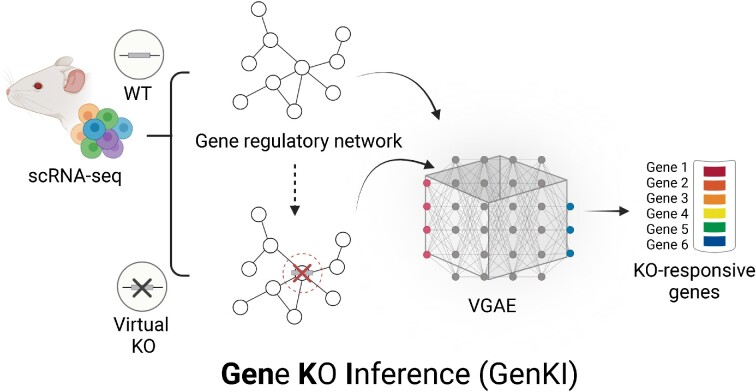

## INTRODUCTION

Gene perturbation experiments are a proven powerful approach to elucidate the role of a gene in a biological process. Commonly used designs include gene knockout (KO) experiments with genetically altered animals and CRISPR gene perturbations. In a KO experiment, the function of a target gene is inferred by contrasting phenotypes between KO and wild-type (WT) animals and then identifying their differences. Often, gene expression profiles serve as a quantitative phenotype at the molecular level (1). The recent advent of single-cell RNA sequencing (scRNA-seq) (2) allows the transcriptomic information from tens of thousands of cells to be gathered in parallel, and thus it greatly improves cellular phenotyping resolution. It has become a powerful method for molecular phenotyping and comparison in KO experiments.

Conventional KO experiments, often requiring significant amounts of experimental and animal resources, are labor-intensive and time-consuming (3). Recently developed techniques such as Perturb-seq (4) combine CRISPR perturbations and scRNA-seq to perform genetic screens, allowing gene function to be studied in many cells in a massively parallel manner. Nevertheless, the creation of large-scale CRISPR libraries presents a major technical challenge. For these reasons, computational tools serve as a possible alternative solution to facilitate or guide the experimental design through in-silico screening of perturbation responses. Such a computational tool would reduce the need for experimental measurements.

Indeed, several such computational tools (5–8) have been developed (Table 1). With only one exception—scTenifoldKnk (8), all these tools require extensive input data sets including outcomes of perturbation experiments or data from other modalities. scTenifoldKnk is the only protocol that does not require such expensive input data sets. Instead, it merely requires scRNA-seq data from the WT samples as its input and considers information from the gene regulatory network (GRN). The working principle of scTenifoldKnk is to simultaneously project WT and virtual KO single-cell gene regulatory networks (scGRNs) to a joint low-dimensional space and then calculate the projection differences of genes. However, the inference of scTenifoldKnk entirely relies on the WT scGRN, which is constructed using principal component (PC) regression from the WT scRNA-seq data. It is known that constructing high-quality scGRNs is technically challenging with respect to the presence of heterogeneous sources of noise (9). Also, a fully connected scGRN computed by the regression-based method may not correspond to real biological processes (10). A method that takes full advantage of scRNA-seq expression data and tolerates imperfect scGRN in a robust and unbiased manner is still lacking.

**Table 1.** Summary of existing virtual KO methods and feature comparison with GenKI

Name	Input data required	Method	Supervised / unsupervised	Description	Reference
scGen	scRNA-seq (WT and KO samples)	Transfer learning	Supervised	Train a variational autoencoder that learns to generalize the response of the cells in the training set of perturbations	(5)
CPA	scRNA-seq (KO samples)	Generative modeling	Supervised	Train an autoencoder with adversarial that decomposes the data into a collection of embeddings associated with the cell type, perturbation, and other external covariates to study combinatorial genetic perturbation	(6)
CellOracle	scRNA-seq and scATAC-seq (WT sample)	Graph-based modeling	Unsupervised	Simulate gene expressions in response to transcription factor (TF) perturbation by signal propagation through an inferred gene regulatory network	(7)
scTenifoldKnk	scRNA-seq (WT sample)	Manifold alignment	Unsupervised	Simultaneously project inferred WT and virtual KO gene regulatory networks to a joint low dimensional space	(8)
GenKI	scRNA-seq (WT sample)	VGAE	Unsupervised	Train a VGAE model that learns the latent gene representations of WT sample and virtually construct a virtual KO counterpart to discern similarity	This study

Here, we present GenKI (**Gen**e **K**O **I**nference), a virtual gene KO tool based on a variational graph autoencoder (VGAE) (11). GenKI simultaneously learns latent representations of scRNA-seq gene expression data of WT samples and the underlying scGRN responsible for observed phenotypes. The highly compressed representations of genes are then used for the subsequent inference. The scGRN can be constructed using the input gene expression data. GenKI propagates the transcriptomics information in the network during training and compares the WT data (including the expression data matrix and the scGRN) with its virtual KO counterpart to predict KO-responsive genes—i.e. genes functionally associated with or linked to KO gene. As a *de novo* inference tool, GenKI identifies KO-responsive genes without requiring prior knowledge of gene regulation or biological mechanisms.

The remainder of this paper is structured as follows: we first present an overview of the GenKI workflow and then compare its inference performance to several benchmarks using simulated data. Following these steps, we use publicly available scRNA-seq data sets (Supplementary Table S1) to predict KO-responsive genes and compare enriched functions of them with those introduced and validated in the original studies, to highlight the performance of GenKI in real-data applications. Next, we compare GenKI to the differential expression (DE) analysis. Finally, we study the robustness and scalability of GenKI.

## MATERIALS AND METHODS

### Simulated data sets and evaluation

The predefined GRNs were obtained from the GitHub repository of SERGIO (12) https://github.com/PayamDiba/SERGIO. The simulated data sets contained 100, 400, and 1200 genes (all containing 2700 cells), respectively. Edges in the predefined GRNs were treated as the ground truth. A random classifier that ranks genes by probabilities randomly drawn from a uniform distribution between 0 and 1, a classifier that ranks genes by the Pearson correlation with the KO gene, and scTenifoldKnk, which ranks genes by FC (used for the chi-squared test), were included for benchmarking purpose. For each data set, we randomly selected a target gene with more than ten edges and virtually knocked it out using GenKI and the other three benchmarks independently. Each run outputs a gene list with scores assigned by each method. *Roc_auc_score* and *average_precision_score* function from the Python package sklearn (v.1.1.1) were used to compute the Area Under Receiver Operating Characteristic (AUROC) and the average precision (AP) at each run for each method. We repeated the procedure above ten times for each data set. The simulated BEELINE (13) data sets were downloaded from Zenodo. GSD is the largest curated reference data set of BEELINE containing 19 genes and 2000 cells. Its underlying GRN was used to replace the GRN construction step in this evaluation. Since the ground truth GRN was known, we divided genes into two groups based on their shortest path to the KO gene, with the close neighbors group containing all genes within the two-hop neighborhood of the KO gene and the distant neighbors group containing all other genes. To compare the inference power of GenKI and scTenifoldKnk, we virtually knocked out each gene iteratively and obtained the scores of all the genes computed by both methods. For each method, we used the Wilcoxon Rank Sum test to quantify the difference in scores between the two groups of genes. A lower *p-*value indicates a larger difference, thus implying greater inference power of the method for detecting KO-responsive genes.

### Processing of real data sets

The specifics and source of real scRNA-seq data sets used in this paper can be found in Supplementary Table S1. We performed regular preprocessing for all scRNA-seq data sets using Seurat (v.4.0.2) package (14). We first performed log normalization using the *NormalizeData* function. Highly variable genes were selected using the *FindVariableFeatures* function (selection.method = ‘vst’) and by default, the top 3000 highly variable genes were included in subsequent analyses. We then standardized the data by the *ScaleData* function, and the resulting transformed data served as the gene expression profile for the GenKI input. Cell annotations from original studies were retained and used if provided.

### Gene regulatory network construction

We constructed scGRNs using the PC regression method which was first proposed in scTenifoldNet (15). Let ${\boldsymbol{X}}\in{\mathbb{R}}^{p \times n}$ represent the scRNA-seq gene expression matrix of the WT samples, which contained gene expression levels for $p$ genes in $n$ cells. We used the PC regression method to build the scGRN denoted with its adjacent matrix ${\boldsymbol{A}}$. Specifically, each time one gene was selected as the response variable, while the remaining genes served as explanatory variables. Principal component analysis (16) was performed on the explanatory variables, and then we regressed the response variable on the first $d$ leading PCs, where $d \ll n$. Next, we transformed the obtained regression coefficients of the $d$-leading PCs into the coefficients of the original explanatory variables, which should reflect the interaction strengths between the response gene and all other genes. In the final step, we assembled the coefficients of $p$ regression models into a $p \times p$ adjacency matrix ${\boldsymbol{A}}$, where the $( {i,\ j} )$ entry represents the regression coefficient of the $i$-th gene on the $j$-th gene. Therefore, ${\boldsymbol{A}}$ accumulates the interaction strength between each pair of genes.

Note that the output of this PC regression method is a fully connected scGRN, in which some links between genes might not correspond to real biological interactions, as in general, there are very few connections between TFs and genes (10). Therefore, for such an scGRN, we assumed that the edge is activated if the absolute value of its weight is greater than a certain threshold, i.e. edges with a greater weight are more likely to be the true regulatory relationships between genes than those with a lower weight. The average absolute weight between TF-target gene pairs constructed scGRNs was indeed significantly greater than that between random gene pairs, as described in (15). Based on these findings, for a particular scGRN, we filtered edges and, by default, conservatively only kept the top 15% of edges. A more thorough evaluation of the cutoff selection can be found in Supplementary Figure S1, which shows a heatmap of Spearman correlation coefficients between scores of Kullback–Leibler (KL) divergence given by GenKI across four different cutoffs. Within an optimal range of the cutoff, the ranking results given by GenKI were found to be highly consistent. However, we contend that extremely conservative choices of the cutoff would overlook potential links. Notably, we allow users to modify this default setting to accommodate their own biological scenarios. For example, those who believe their gene regulatory networks are scale-free are encouraged to use the poweRlaw package (17) to determine the best-fit threshold. Next, we converted the scGRN into an adjacent Boolean matrix as the input requested for the VGAE model of GenKI. As a result, although obtained without any information on TFs and their targets or knowledge of regulatory elements, these remaining edges could be deemed biologically responsive. By abuse of notations, we still denoted this new scGRN as ${\boldsymbol{A}}$ and we referred to it as the thresholded scGRN for later use. Although the filter step removed potential false positive edges, it inevitably introduced false negative findings, i.e. missing some truly connected edges. Therefore, we treated this thresholded scGRN as an incomplete network, and our goal was to reconstruct an scGRN from this incomplete network to learn the latent embeddings of nodes, namely, genes in our setting. This can be interpreted as a transductive link prediction task (18). Alternatively, users can supply their own GRN at this step to replace the PC regression-derived network.

### VGAE model

The VGAE model used in GenKI is similar to the framework described in (11). It is made up of a two-layer graph convolutional network (GCN) encoder and an inner product decoder. We utilized a two-layer GCN architecture because deeper graph convolutional networks are prone to over-smoothing (19). Recall that ${\boldsymbol{X}}$ is the gene expression matrix and ${\boldsymbol{A}}$ is the adjacent matrix, and we denoted the normalized adjacent matrix as ${\boldsymbol{\tilde{A}}} = {{\boldsymbol{D}}}^{ - \frac{1}{2}}{\boldsymbol{A}}{{\boldsymbol{D}}}^{ - \frac{1}{2}},$ where ${\boldsymbol{D\ }} = \ diag( {{d}_{11},\ {d}_{22}, \ldots ,\ {d}_{pp}} )$ is a diagonal matrix with entries ${d}_{ii} = \mathop \sum \limits_{{\rm{i}} = 1}^{\rm{p}} {A}_{ij}$, where ${\boldsymbol{A}}_{ij}$ is the ($i,j$)-th entry of the matrix $\boldsymbol{A}$. Then, the two-layer GCN is defined as:


$$\begin{equation*}GCN\left( {{\boldsymbol{X}},{\boldsymbol{A}}} \right) = {\boldsymbol{\tilde{A}}}ReLU\left( {{\boldsymbol{\tilde{A}X}}{{\boldsymbol{W}}}_0} \right){{\boldsymbol{W}}}_1\end{equation*}$$

where $ReLU( {\boldsymbol{x}} ) = {\rm{max}}( {0,\ {\boldsymbol{x}}} )$ is the activation function introduced in the first GCN layer, and ${{\boldsymbol{W}}}_0$ and ${{\boldsymbol{W}}}_1$ are parameters of the neural networks. We assumed that the data were generated by certain random processes involving an unobserved latent continuous random variable ${\boldsymbol{Z}}$. Let $p( {\boldsymbol{Z}} )$ be the prior distribution of ${\boldsymbol{Z}}$, for which we chose a bivariate Gaussian distribution for convenience. For the encoder part, we introduced a recognition model $q( {{\boldsymbol{Z}}{\rm{|}}{\boldsymbol{X}},\ {\boldsymbol{A}}} ) = \mathop \prod \limits_{i = 1}^p q({{\boldsymbol{z}}}_i|{\boldsymbol{X}},\ {\boldsymbol{A}})$, where $q( {{{\boldsymbol{z}}}_i{\rm{|}}{\boldsymbol{X}},\ {\boldsymbol{A}}} ) \sim \mathcal{N}( {{\mu }_i,\ {{\rm{\Sigma }}}_i} )$, ${{\rm{\Sigma }}}_i = diag( {\sigma _{i1}^2,\sigma _{i2}^2} )$ is a diagonal covariance matrix and


$$\begin{equation*}{\boldsymbol{\mu }} = {\left( {\mu _1^T,{\boldsymbol{\ }} \cdots ,\mu _p^T} \right)}^T = GC{N}_\mu \left( {{\boldsymbol{X}},{\boldsymbol{\ A}}} \right),\end{equation*}$$

$$\begin{equation*}log\left( {{\bf \Sigma }} \right) = log\left( {\left( {\sigma _1^2,\ \cdots ,\ \sigma _p^2} \right)} \right) = GC{N}_{{\sigma }^2}\left( {{\boldsymbol{X}},\ {\boldsymbol{A}}} \right),\end{equation*}$$

where $\sigma _i^2 = {[ {\sigma _{i1}^2,\sigma _{i2}^2} ]}^T.$ For the decoder part, we used the inner product to reconstruct the scGRN ${\boldsymbol{A}}$ by


$$\begin{equation*}P\left( {{{\boldsymbol{A}}}_{ij} = {{\boldsymbol{A}}}_{ji} = 1} \right)\ = \ sigmoid\left( {{\boldsymbol{z}}_i^T{{\boldsymbol{z}}}_j} \right).\end{equation*}$$

Here, by abuse of notations, ${{\boldsymbol{z}}}_i$ is the latent representation of the $i$th gene.

For any two distribution functions $p$ and $q$, let $KL( {p\parallel q} ) = \smallint p( x )log\frac{{p( x )}}{{q( x )}}dx$ be the KL divergence between $p$ and $q$. The objective of the VGAE model is to maximize the evidence lower bound (ELBO):


$$\begin{equation*}\mathcal{L} = {\mathbb{E}}_{q\left( {{\boldsymbol{Z}}{\rm{|}}{\boldsymbol{X}},\ {\boldsymbol{A}}} \right)}\log p\left( {{\boldsymbol{A}}{\rm{|}}{\boldsymbol{Z}}} \right) - \beta \cdot KL\left( {q\left( {{\boldsymbol{Z}}{\rm{|}}{\boldsymbol{X}},{\boldsymbol{A}}} \right)\parallel p\left( {\boldsymbol{Z}} \right)} \right),\end{equation*}$$

where $\beta$ is an adjustable hyperparameter that balances the independent constraints and reconstruction accuracy. Notice that here we adapted the loss from beta-VAE (20) and $\mathcal{L}$ would represent the standard ELBO when $\beta \ = \ 1$.

### Hyperparameters, metrics and implementation

We randomly split the edges of a Boolean scGRN into three data sets for training (75%), validation (5%), and testing (20%). We labeled them as positive edges. Equal numbers of negative edges, composed of a set of fake edges not presented in the scGRN, were sampled for data balancing purposes. We used AUROC and AP to evaluate the model performance. We expected positive edges to have higher interaction probabilities compared to negative edges. Thus, the higher value of AP or AUROC would indicate better performance of training. To tune the hyperparameters, we performed random hyperparameters search of 100 trials by using the Tune module from the Python package Ray (21) (v.1.13.0). Specifically, the logarithm base 10 of hyperparameter $\beta$ was sampled from a uniform distribution from {–5, –4, …, –1}, the learning rate was sampled from a uniform distribution from {–4, –3, …, –1}, and the weight decay of optimizer was sampled from a uniform distribution from {–7, –6, …, –3}. To make our sampled hyperparameters more accurate, we multiplied each one by a scale factor randomly selected from integers 1 to 9. For each set of hyperparameters, we evaluated the model performance on the validation set and selected the hyperparameter set with the best performance based on the metrics AUROC and AP. Based on our experimental results, we set $\beta$ of 1E-4 and weight decay of 9E-4 for all the data sets, and set learning rate of 7E-4 for the microglia, lung, intestine data set, 5E-3 for the COVID-19 data set. The maximum iteration number was set to 100, and early stopping was added when AP reached the maximum and began to decrease. The Adam optimizer (22) was used for all the trainings, and Xavier initialization (23) was used to initialize all the weights.

### Determination of the rank of KO-responsive genes

After training the VGAE model using the WT data, for each fixed gene $g$, we obtained its latent distribution *N*($\hat{\mu}_{g}$, $\hat{\sigma}_{g}^2$), where $\hat{\mu}_{g}$ and $\hat{\sigma}_{g}^2$ were latent mean and covariance fitted by the VGAE model. We next fed the trained VGAE model with the virtual KO data and obtained the latent distribution of the $g$-th gene for the KO samples. Then, we calculated the KL divergence between these two normal distributions. The procedure was repeated for all genes. The top 5% of genes ranked by the KL divergence were preserved. Instead of using the raw ranks, we proposed a bagging-based method to improve the stability and accuracy of our inference. Specifically, each time we permutated the cell order of the WT gene expression matrix and obtained its corresponding virtual KO data. Without training a new model, we fed this pair of permutated WT and virtual KO data into our fitted VGAE model, calculated the KL divergence value for each gene, and bagged the top 5% of genes. We repeated this procedure 1000 times and compiled the genes which were bagged more than 95% times as KO-responsive genes.

### Benchmarking GenKI’s tolerance to random noise in gene expression profiles

To show the robustness of our method, we generated random noise in the log space, added it to gene expression profiles, and evaluated the training performance of GenKI. Specifically, for gene $i$ in cell $j$, the regenerated expression $x_{i,j}^{\prime}$ was defined as:


$$\begin{equation*}\frac{{x_{i,j}^{\prime}}}{{{x}_{i,j}}} = {2}^\gamma \end{equation*}$$

where $\gamma\sim\mathcal{N}(0,\,\sigma^{2})$ and ${x}_{i,j}$ represents the original expression. The fold change $\gamma$ was used to approximate the noise level, which followed the normal distribution $\mathcal{N}(0,\,\sigma^{2})$, whereas different $\sigma$ values would result in different levels of random noise. We conducted 30 independent runs with random splits of the data set at different noise levels.

### Gene function annotation and function enrichment tests

Enrichr (24) with default setting was used for gene functional enrichment analyses. The protein-protein interaction enrichment tests were performed using the web tool of the STRING database (25). In the STRING network plots, isolated nodes were removed, and only edges labeled with confidence greater than the medium level were retrieved and shown. Enrichment *p-*values, which indicate whether input proteins have more interactions among themselves than what would be expected for a random set of protein-coding genes of the same size and degree distribution drawn from the genome, were computed with the default setting.

### Prediction of KO gene's expression from WT cells with linear regression

For the microglia data set, a simple multivariate linear regression model was applied to evaluate the relationship between the KO gene Trem2 and other KO-responsive genes. Specifically, microglia cells’ Trem2 expression profile was used as the response variable and the expression profiles of other genes as explanatory variables. The adjusted ${R}^2$ (coefficient of determination) was used to quantify how much variance of the KO gene can be explained by the other KO-responsive genes. In comparison, an equal number of the KO-responsive genes were randomly sampled as explanatory variables, and their ${R}^2$ was also calculated. This evaluation was repeated 30 times with different splits of the data set and random gene selections.

### Differential gene expression analysis

DE analysis was performed using Scanpy (26)(v.1.9.1) function *rank_genes_groups* with the Wilcoxon rank-sum test. All parameters were set to default. Adjusted *p-*values were obtained after the Benjamini–Hochberg adjustment (27). DE genes were determined based on the condition of adjusted *p-*value < 0.05 and absolute log2(fold change) > 0.25. DE ranks of the DE genes were determined based on their adjusted *p-*value. To examine the expression level changes, for each data set, the KO-responsive genes and an equal number of randomly chosen unperturbed genes were used and their fold change (FC) of WT/KO was calculated. The absolute log2-transformed FC values of the KO-responsive genes and the unperturbed genes were used to perform the one-sided t-test.

## RESULTS

### The GenKI framework

**Figure 1.**
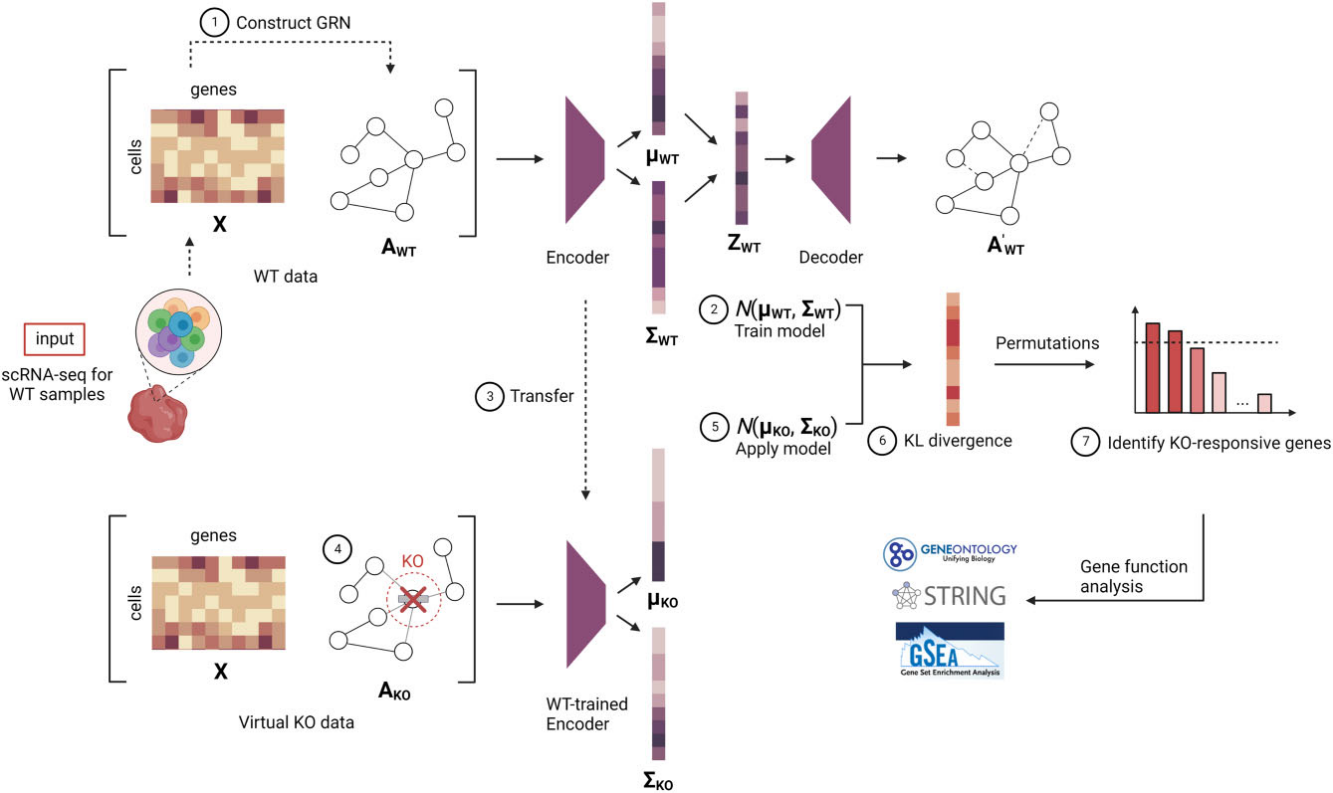
The pipeline contains seven steps: (1) construction of WT scGRN, (2) training VGAE model, (3) transfer the trained VGAE model, (4) construction of virtual KO data, (5) latent embeddings of WT and virtual KO data, (6) calculation of KL divergence, and (7) identification of KO-responsive genes for function annotation and analysis.

The framework of GenKI is depicted in Figure 1. The pipeline starts with a single input, that is, the scRNA-seq gene expression matrix from WT samples of interest. For each virtual KO application, GenKI first constructs an scGRN from the WT gene expression data. The WT gene expression data matrix and the constructed WT scGRN are then used as input of WT data to train a VGAE model, which is a two-layer GCN encoder with an inner product decoder. The latent embedding of each node is defined to follow a bivariate Gaussian distribution. After training, the latent representations of genes under the WT setting are collected and the model with its weights is transferred. Next, to generate virtual KO data, the WT data is ‘copied’. From the WT scGRN copy, the KO gene—i.e. the gene being knocked out for functional study—is virtually deleted. The deletion is achieved by setting the weight of all edges from and to the KO gene to zero. After the virtual deletion, the virtual KO data is generated, while the original WT scGRN remains untouched. The transferred model is fed with the virtual KO data to obtain the latent representations of genes under the KO setting. Two parameters, mean and covariance of each gene's latent distribution from the WT and KO settings are then collected to calculate the KL divergence between these two distributions. The higher the KL divergence value of a gene, the greater the impact of the KO on the gene. Finally, a bagging-based method is used to determine genes that tend to be significantly perturbed by the deletion of the KO gene. The enriched functions of these significantly perturbed genes (i.e. KO-responsive genes) are used to give prediction of the KO gene functions.

### Performance of GenKI with simulated data

**Figure 2.**
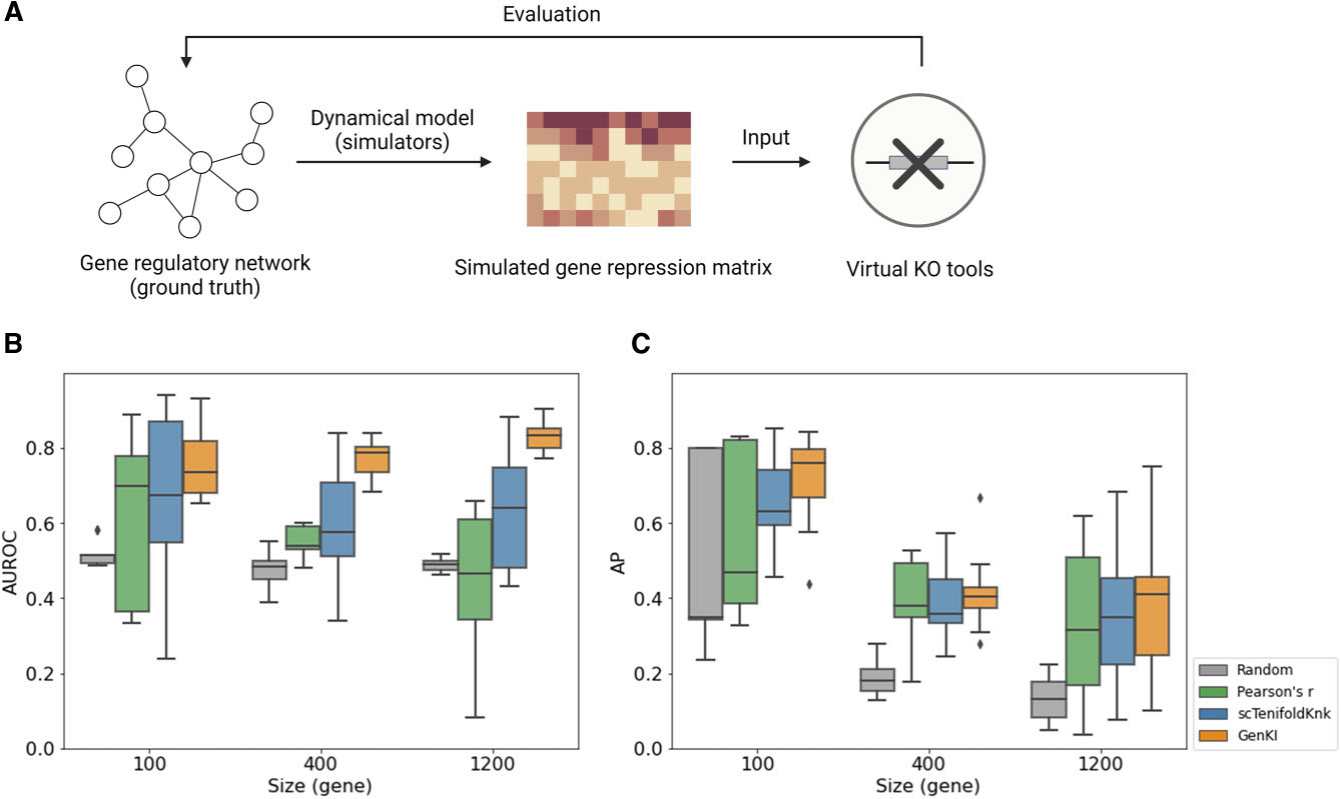
Three methods were included in the comparison including scTenifoldKnk and two baseline predictors, which are based on random rankings and Pearson's correlation, respectively (Materials and Methods). (**A**) The procedure of assessment of virtual KO tools using simulated data sets. (**B**) The levels of AUROC of virtual KO experiments using three simulated SERGIO data sets. (**C**) The levels of AP of virtual KO experiments using three simulated SERGIO data sets. Size represents the number of genes in each data set.

We used simulated data to evaluate the performance of our method (Figure 2A). To do so, we generated scRNA-seq data sets of different sizes (2700 cells with 200, 400, and 1200 genes, respectively) using single-cell expression simulator SERGIO (12). SERGIO’s simulations were guided by predefined GRNs; therefore, the simulated scRNA-seq data sets had their underlying GRNs. Knowing these ground truths GRNs facilitated the performance evaluation of virtual KO methods, as genes linked with the KO gene were supposed to be perturbed by the KO and more likely to be KO-responsive genes. A good virtual KO tool should preferably identify those genes linked with the KO gene in the given GRN. For each of the simulated data sets, we applied GenKI and three other benchmarking methods, including scTenifoldKnk, with the same KO genes being knocked out (Materials and Methods). All the methods produced a ranked list of KO-responsive genes. Figure 2B shows the levels of AUROC for GenKI and other benchmarking methods. Figure 2C shows the levels of AP resulted from the same KO genes. Three additional ROC curves as examples of virtual KO experiments performed by GenKI and scTenifoldKnk for each data set are presented in Supplementary Figure S2. We found that GenKI outperformed all the other benchmark methods, including scTenifoldKnk, across all the data sets evaluated. We believe this is because GenKI incorporates information from both the gene expression matrix and GRN.

To demonstrate that GenKI learns higher-order neighborhood information from the underlying GRN through the VGAE model, which contributes to its greater performance than scTenifoldKnk, we systematically knocked out each of the 19 genes in the GSD network of BEELINE (13). In each virtual KO experiment, we obtained the perturbation scores of all genes. For a given KO gene, we used the Wilcoxon rank sum test to compare the difference in perturbation scores between the KO gene's two-hop neighbor genes and all the other distant genes (Materials and Methods). A smaller *p-*value indicates a greater inference power of the method for differentiation between these two groups. It is rational to expect that close neighbor genes have high perturbation scores. Compared to scTenifoldKnk, as expected, *p-*values obtained in GenKI are significantly lower (Supplementary Figure S3, Wilcoxon Rank Sum test, *p-*value < 0.05). This is attributed to manifold alignment in scTenifoldKnk only keeps track of the similarities between genes in the first-order neighborhood of GRN, while GenKI’s two-layer GCN looks at similarities between genes up to the second-order neighborhood. This simulation study using the BEELINE network data also demonstrated that GenKI can take user input GRN as an optional rather than reconstructing GRN by its own.

### Real-data GenKI analysis recapitulates findings of the trem2 KO experiment

**Figure 3.**
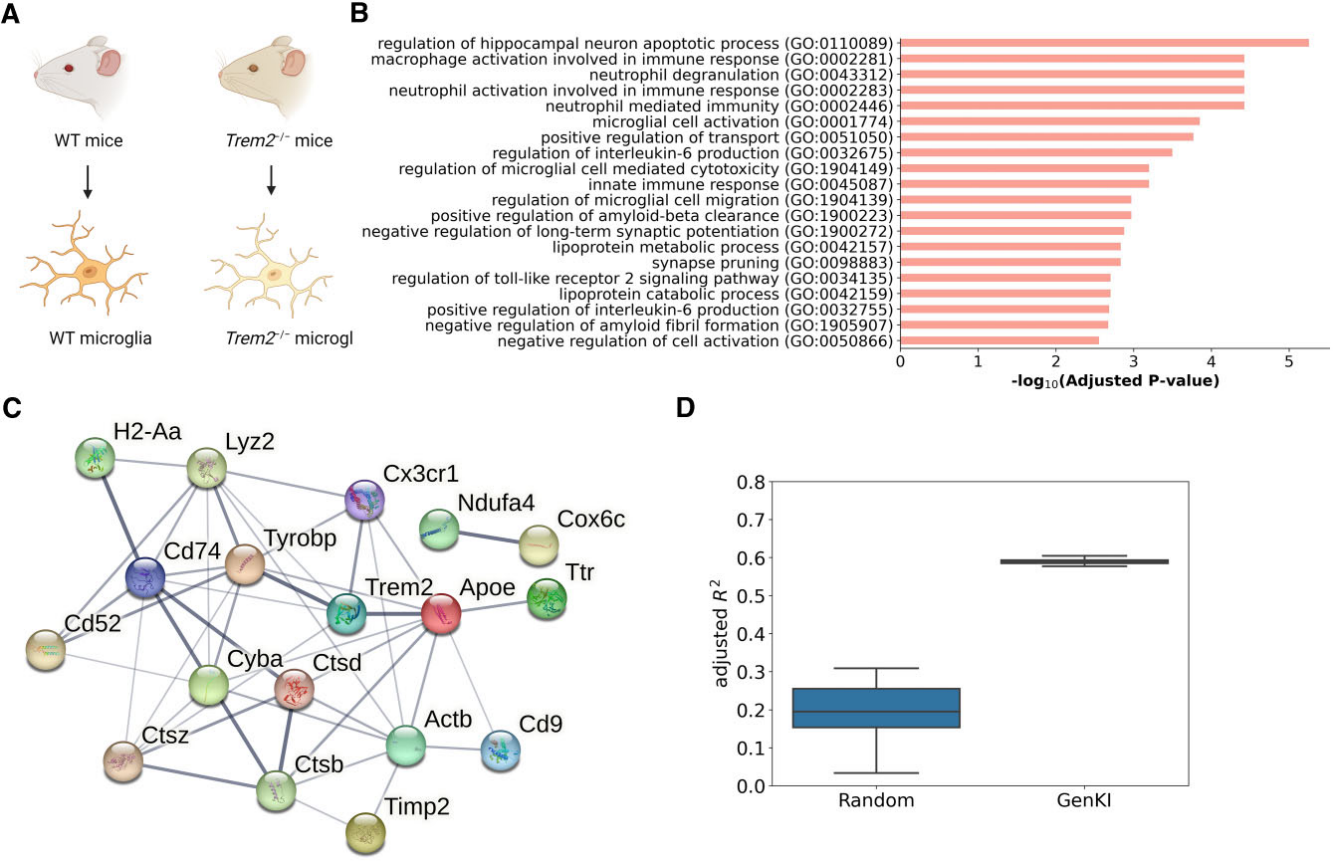
Trem2-KO responsive genes inferred by GenKI. (**A**) Illustration of Trem2-KO experiment generating the microglia data set. (**B**) GO terms significantly enriched in functions of Trem2-KO responsive genes. The –log_10_-transformed adjusted *p-*value indicates the strength of enrichment for each term. (**C**) STRING network of Trem2-KO responsive genes. Edge thickness indicates the strength of data support. (**D**) Adjusted *R*^2^ score of the regression of expression levels by setting the Trem2 as a response variable and other KO-responsive genes as explanatory variables, compared to that of randomly selected genes as the explanatory variables.

GenKI, as a virtual KO tool, is expected to recapitulate the overall discoveries of real KO experiments. To validate its performance, we applied GenKI to several publicly available scRNA-seq data sets. The first data set was from the KO experiment conducted by Nugent *et al.* (28), in which scRNA-seq was performed with microglial cells isolated from Trem2^+/+^ and Trem2^−/−^ mice (Figure 3A). The study reported that Trem2 upregulates apolipoprotein E (Apoe) and other genes involved in cholesterol transport and metabolism, causing robust intracellular accumulation of a storage form of cholesterol upon chronic phagocytic activities (28). Trem2 is also known to regulate the expression of genes associated with cell damage response, lysosome and phagosome function, Alzheimer's disease, and oxidative phosphorylation (29). With this data set, we used the WT gene expression profile of 648 microglial cells as the input for GenKI and fed it along with the constructed scGRN to the VGAE model of GenKI.

We first evaluated the robustness of our model before performing prediction. The model robustness evaluation was performed to test the tolerance of the model by artificially adding different levels of random noise to the WT gene expression profile (Materials and Methods). A robust model would correctly capture the latent embeddings of genes, and thus more confidence for the inference regarding differences between WT and virtual KO samples. AUROC and AP were used to evaluate the reconstruction performance of the model. As shown in Supplementary Figure S4, our model was not compromised by high levels of noise ($\sigma$ = 1.5), indicating the robustness of GenKI to the technical noise that naturally existed in the scRNA-seq data. We observed poorer performance under the conditions of very high levels of noise ($\sigma$ ≥ 3), which was expected as highly noisy gene expression profiles would mislead the training, and thus, the model could not be generalized to the testing data set. These results also indicated the lower bound of noiseless gene expression information needed to correctly reconstruct the scGRN and eventually infer the latent embeddings of genes.

After the model robustness evaluation, we then trained the model and performed the virtual KO experiment. Specifically, we virtually knocked out Trem2 by removing all its edges in the scGRN of microglial cells and compared profiles of genes in the latent space between WT and virtual KO samples using KL divergence (Materials and Methods). The results of the analysis showed that 20 genes, including Trem2 itself, were detected as Trem2-KO responsive genes (Supplementary Table S2). Trem2 was ranked at the top of the KO-responsive genes, followed by Ctsd, the gene associated with lysosomal dysfunction (30), and Apoe, the key lipid transporter gene expressed in both the central nervous system and the periphery (31). Pathway enrichment analysis based on Enrichr (24) showed that Trem2-KO responsive genes were enriched with genes associated with *interleukin-2 signaling pathway*, *lysosome*, and *Alzheimer's disease* (Supplementary Table S3). Gene ontology (GO) enrichment analysis further ranked several enriched terms, including *macrophage activation involved in immune response* and *lipoprotein metabolic process*, on the top (Figure 3B and Supplementary Table S4). By modulating the macrophage transcriptome in adipose tissue, Trem2 was found to regulate blood cholesterol metabolism in obese mice, thereby indicating a connection between Trem2 and lipid metabolism (32). The overall results of our enrichment analyses revealed these functions of Trem2 with consistency. In addition, the Trem2-KO responsive genes were found to be biologically connected, as shown by the STRING interaction network (25) (Figure 3C, *p*-value < 0.01, STRING interaction enrichment test). Note that links in STRING interaction networks represent functional associations between genes. These associations include direct regulations as well as indirect interactions between genes or their products. Thus, our results suggest abundant functional connectivity between KO-responsive genes.

Next, we investigated whether Trem2’s measurable gene expression was intrinsically interpreted by other KO-responsive genes. Indeed, the variance of Trem2 expression across cells could be substantially explained by the remainder of the KO-responsive genes (Figure 3D). We fitted a multivariable linear regression model by setting Trem2 as the response variable (Materials and Methods) and found that when using KO-responsive genes as explanatory variables, the adjusted ${R}^2$ of the model was significantly higher than when using an equal number of randomly selected genes as explanatory variables (*p-*value < 0.01, one-sided t-test). This finding suggests the KO gene and its KO-responsive genes predicated by GenKI tend to be transcriptionally associated.

Finally, we showed that one could not simply obtain the ranked gene list inferred by GenKI to identify KO-responsive genes using naïve network analysis metrics. We presented that, as an example, the KO-responsive genes could not be simply inferred either from ranking their gene expression or edge weight associated with the KO gene Trem2 in the inferred scGRN (Supplementary Figure S5). The GenKI model nonlinearly learns both gene expression and edge weight information and infers from compressed embeddings of genes that it has learned. Thus, it ranks and infers the perturbed genes in a more comprehensive way than ranking methods based on any single observable property.

Collectively, our results shed light on Trem2-related functions by annotating the perturbed genes following its deletion. We showed that the inferred genes were functionally connected and, more importantly, predicted functions were consistent with those reported in the Trem2 studies.

### Real-data GenKI analysis recapitulates findings of the nkx2-1 KO experiment

NK homeobox 2–1 (Nkx2-1) is highly expressed in lung epithelial cells and plays a crucial role in alveolar type 1 (AT1) cell development and maintenance (33). We collected the second scRNA-seq data from an *in vivo* KO experiment performed with lung epithelial cells of AT1 isolated from WT and Nkx2-1^−/−^ mice. The study reported that the Nkx2-1 knocked-out AT1 cells lost their characteristics and abnormally turned into gastrointestinal fate (34). The study concluded that without Nkx2-1, developing AT1 cells lose three defining features—molecular markers, expansive morphology, and cellular quiescence—leading to alveolar simplification and lethality.

With this data set, we used the WT gene expression profile of 624 AT1 cells as the input for GenKI and virtually knocked out Nkx2-1 following the methods described above. The GenKI analysis discovered 82 KO-responsive genes (Supplementary Table S5). The KO gene, Nkx2-1, topped the gene list, followed by 13 marker genes of AT1 and AT2 cells offered by PanglaoDB (35), consistent with their downregulation in the Nkx2-1 mutant cells from the bulk RNA-seq experiment introduced in the original study. Previous research (36–39) discovered that Nkx2-1 binds to a group of AT1 cell-specific genes that regulate the cytoskeleton, membrane composition, and extracellular matrix. We found that Pdlim1, Clic5, Tuba1a, Krt8, Actn4, and Clu, which encode cytoplasmic proteins associated with the cytoskeleton, were highly ranked in our list. Ctsh, a gene involved in epithelial tube branching and lung morphogenesis (40), and a great number of genes related to membrane composition, such as Anxa1, were also observed among the KO-responsive genes. Two other significant genes, Napsa and Sftpc, collaborate with Ctsh to perform functions related to the collagen-containing extracellular matrix and alveolar lamellar body. Cldn33, Cldn7, and Epcam, which were shown to be involved in the apical junction complex (41), are in agreement with the observation that mutant AT1 cells form dense microvilli-like structures apically concluded in the original study.

**Figure 4.**
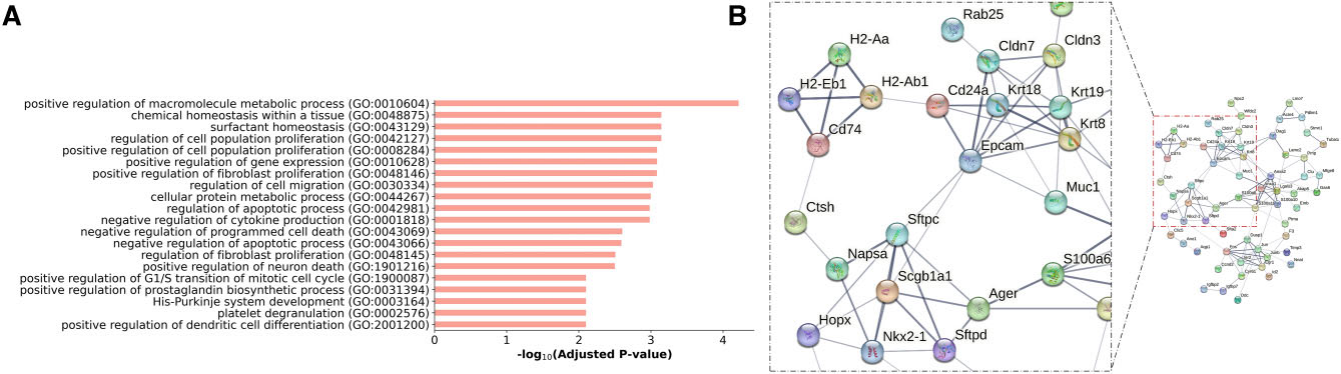
Nkx2-1-KO responsive genes inferred by GenKI. (**A**) GO terms significantly enriched in functions of Nkx2-1-KO responsive genes. The -log10-transformed adjusted *p-*value indicates the strength of enrichment for each term. (**B**) STRING network consists of Nkx2-1-KO responsive genes. The zoomed inset demonstrates a subnetwork module containing the KO gene.

GO enrichment analysis indicates these genes were enriched for functional categories led by *surfactant homeostasis* and *positive regulation of cell population proliferation* (Figure 4A, Supplementary Table S6), suggesting the role of Nkx2-1 in regulating surfactant production and suppressing AT1 cell proliferation validated in the study. HDAC3-dependent TGF-beta signaling is required for proper epithelium expansion and AT1 cell spacing (42,43), disruption of which significantly perturbed 13 genes from the list related to *TGF-beta regulation of extracellular matrix*. Additionally, due to mutant cells undergoing apoptosis, which was validated by staining in the original study, a few terms indicating the apoptotic process were observed. Many other GO terms, which are significant but not shown in Figure 4A, such as *epithelial tube branching involved in lung morphogenesis* and *epithelial cell morphogenesis* demonstrate the conclusion that Nkx2-1 defines the cell morphology of developing AT1 cells. The STRING interaction network of these 82 KO-responsive genes is shown in Figure 4B, suggesting that they tend to be biologically connected with a closely related functional relationship (*p-*value < 0.01, STRING interaction enrichment test).

### Real-data GenKI analysis recapitulates findings of the hnf4a-smad4 double KO experiment

Using two real scRNA-seq datasets in which a single KO gene was knocked out, we have demonstrated the general performance of GenKI. Next, we investigated whether GenKI is able to virtually predict the effects of double KO (DKO). To accomplish this, we obtained a scRNA-seq data set performed with enterocytes isolated from WT and Hnf4a^KO^-Smad4^KO^ mice. The study reported that Smad4 and Hnf4 work together in a feed-forward loop to activate one another's expression and co-bind to differentiation gene regulatory regions. This feed-forward regulatory module supports and maintains enterocyte cell identity. Loss of this regulatory loop could impair enterocyte differentiation and destabilize enterocyte identity. This intersection of signaling and transcriptional regulation provides a framework for understanding the cellular plasticity of the regeneratable tissue (44).

In this experiment, we used the WT gene expression profile of 502 enterocytes as the input for GenKI and virtually knocked out Hnf4a and Smad4 simultaneously. 14 KO-responsive genes were reported by GenKI (Supplementary Table S7). The two KO genes, Hnf4a and Smad4, topped the gene list, followed by regenerating islet-derived 1 (Reg1), a regulator of cell growth that is required to generate and maintain the villous structure of the small intestine (45). Hnf4a regulates intestinal epithelium homeostasis and intestinal absorption of dietary lipids (46). Loss of this gene is likely to disrupt glucose metabolism, which is regulated by intestinal Reg3b (47), another significant gene. Also included was Gcg, a gene that may modulate gastric acid secretion and gastro-pyloro-duodenal activity (48).

**Figure 5.**
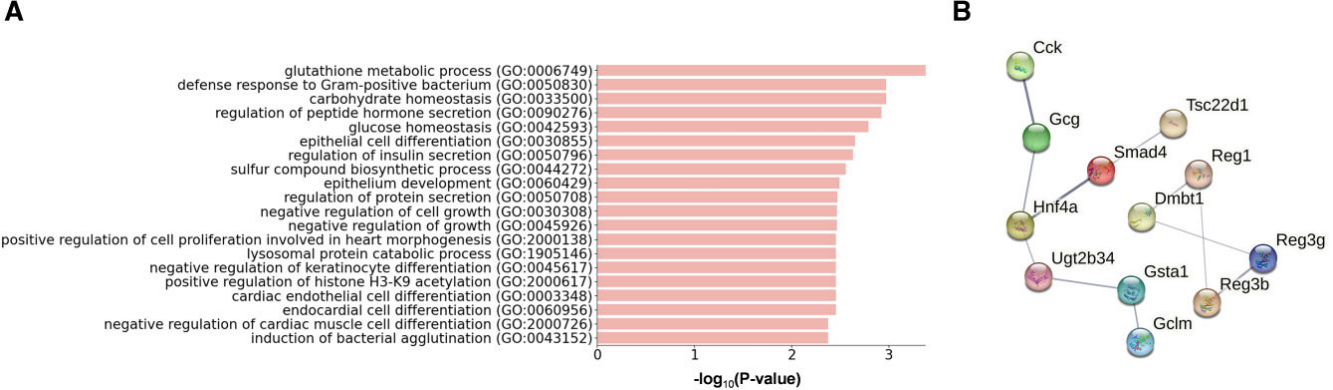
Hnf4a & Smad4-KO responsive genes inferred by GenKI. (**A**) GO terms significantly enriched in functions of inferred Hnf4a-Smad4-KO responsive genes. The –log_10_-transformed *p-*value indicates the strength of enrichment for each term. (**B**) STRING subnetwork consists of Hnf4a-Smad4-KO responsive genes.

Figure 5B depicts the STRING interaction network of these KO-responsive genes. Despite the network being split into two parts under the default setting, we found two disconnected genes, Dmbt1 and Gsta1, were indeed functionally connected—GO enrichment analysis indicates that these two genes were enriched for *epithelium cell differentiation* (Figure 5A, Supplementary Table S8), indicating the loss of enterocytes differentiation after the DKO measure discovered in the original study. Thus, these genes are statistically (*p-*value < 0.01, STRING interaction enrichment test) and biologically linked. Other significant GO terms, such as *negative regulation of cell growth* and *carbohydrate homeostasis* correlated with results of the enterocytes study, have also been illustrated in our analysis. Together, this virtual DKO experiment demonstrates that perturbation effects from multiple KO genes are nonlinearly accumulable and can be recapitulated by GenKI.

### Are KO-responsive genes more likely to be differentially expressed?

We next set out to answer the following question: do KO-responsive genes exhibit differential expression? We first analyzed the expression level changes of predicted KO-responsive genes by comparing them to unperturbed genes across data sets (Materials and Methods). We discovered that the KO-responsive genes predicted by GenKI tend to have greater absolute FC values than unperturbed genes (Supplementary Figure S6, *p-*value < 0.05, one-sided *t*-test). Thus, we came to the conclusion that KO-responsive genes predicted by GenKI are more likely to be differentially expressed.

**Figure 6.**
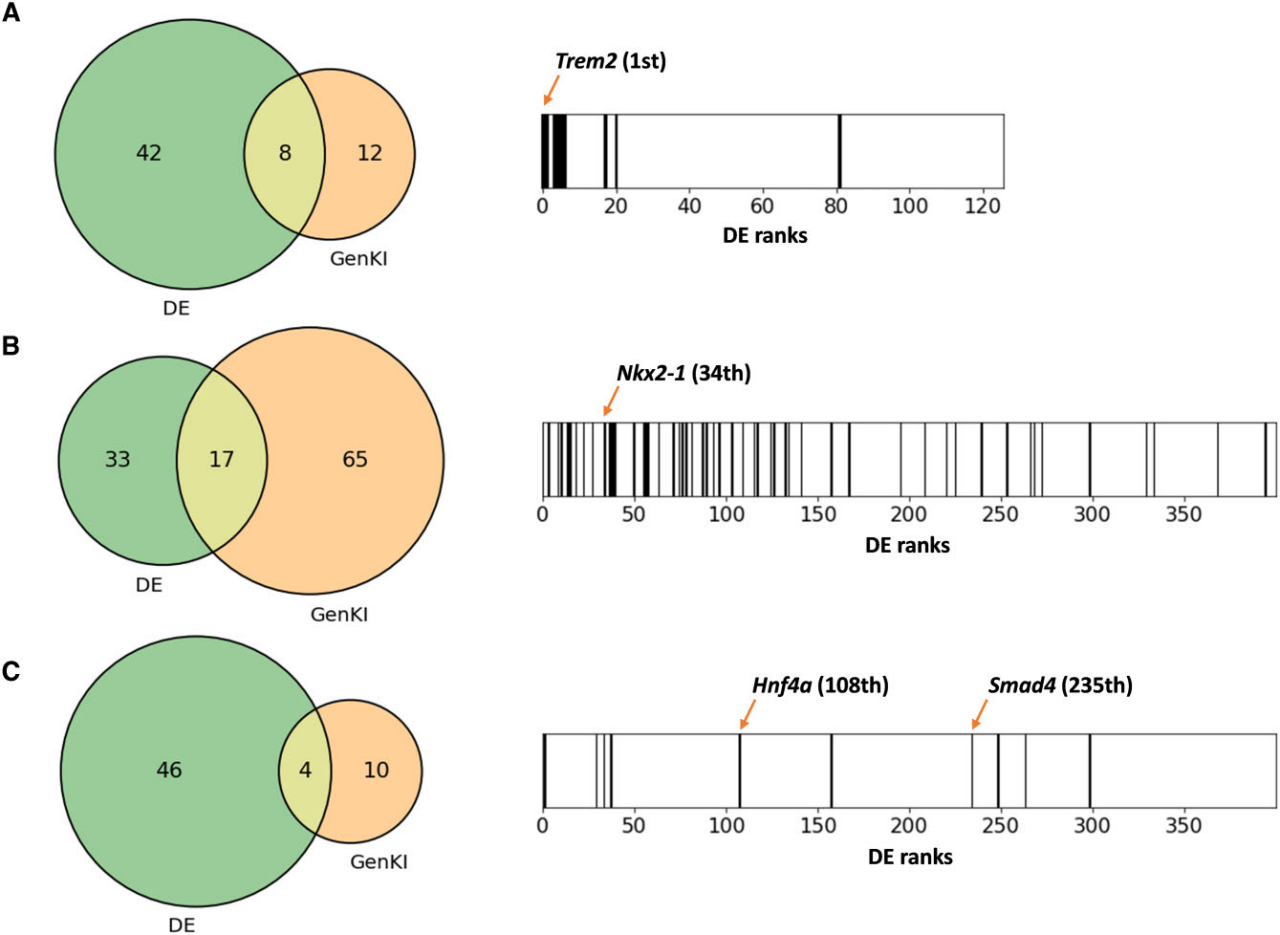
Venn diagrams and barcode enrichment plots showing the intersection and differences between the KO responsive genes given by GenKI and DE genes. Venn diagram and barcode enrichment plot of (**A**) microglia data set, (**B**) lung data set and (**C**) intestine data set. All the numbers of overlapped genes were significantly greater than random expectations (*p-*value < 10E-05, hypergeometric test).

Next we showed that GenKI analysis is different from the DE analysis: KO-responsive genes are not necessarily DE genes. We examined this by comparing the real KO data of each data set to their WT, where 126, 1129 and 1215 DE genes were identified, respectively (Materials and Methods). The overlap between the predicted KO-responsive genes and the top-ranked 50 DE genes in each data set is shown with a Venn diagram in Figure 6 left panel.

The eight overlapping genes of the microglia data set includes Trem2 and other lipoproteins-forming genes like Apoe (Figure 6A, left). The 17 intersection genes of the lung data set contain Nkx2-1, the pulmonary surfactant Sttpc and several AT1 and AT2 cell markers (Figure 6B, left). Thus, GenKI could be used to predict some of the DE genes. In addition, GenKI identified KO-responsive genes that are not ranked highly by the DE method. By using a barcode enrichment plot (Figure 6, right panel), we were able to visualize the exact locations of the KO-responsive genes across the DE ranks, with each black stick denoting a ‘hit’ of the KO-responsive genes. H2-Aa, a recognized DE gene but not ranked highly (82nd shown in Figure 6A, right), is known to function with other genes such as Cd74, Ctsb, and Ctsd in histocompatibility complex (MHC) class II presentation (49). Napsa, which functions together with Nkx2-1 and Ctsh in the processing of pneumocyte surfactant precursors, was likely to be underestimated (763rd, out of scope in Figure 6B, right). The double KO genes Hnf4a and Smad4, which were not included in the intersection of Figure 6C left, weakly ranked 108th and 235th, respectively (Figure 6C, right). These perturbed genes were prioritized by GenKI, whereas the DE analysis did not. GenKI further identified KO-responsive genes that are not DE genes. These genes are likely to be at least as important as the DE genes, if not more. For example, concerning the microglia data set, Ctsd is one leading gene involved in cholesterol metabolism (50), and Cx3cr1 and Tyrobp play an important role in macrophage activation (51–53). All of them were not the DE genes.

Do DE genes appear more adjacent to KO-responsive genes in a scGRN? To answer this question, we performed the STRING network analysis by combining the top-ranked DE genes with the KO-responsive genes using the microglia data set as an example. The outcome is depicted in Supplementary Figure S7, showing that 23 out of 42 DE genes are directly or indirectly linked to the KO genes. That is to say, in this given case, more than half of DE genes might be functionally involved in the perturbed KO gene network identified by GenKI.

Utilizing DE and GenKI analyses in a complementary manner might be a good idea. To illustrate our point, we applied seven different DE analysis methods and settings to the lung data and summarized the number of DE genes detected and their intersection with GenKI-identified genes (Supplementary Table S9). We found that the results of DE analysis were largely depend on what method was selected to use and what fold-change and *p-*value cutoffs were set, and the functional interpretation of the DE analysis results was also depended whether up- and down-regulated genes are pooled together. In general, we found different DE methods with varying model assumptions and thresholds could not converge to a consensus set of DE genes. The number of DE genes and their rankings changed greatly depending on many technical factors as mentioned. Furthermore, most DE methods with default settings produce excessive numbers of DE genes, making downstream functional enrichment analysis difficult and obscuring true signals caused by the perturbation itself to be detected. GenKI, on the other hand, as a method independent of DE methods, provides additional evidence for gene functions. Most of GenKI’s KO-responsive genes overlapped with DE genes regardless of the DE method. With the default setting, GenKI produced fewer significant genes than DE methods, which may improve the interpretability of gene function. In this sense, we are not developing an alternative to DE, but rather a complementary technique that produces more targeted results.

### Real-data GenKI analysis predicts function of key transcriptional factor STAT1

Above we have validated GenKI performance by comparing the inference results to DE genes using three scRNA-seq data sets that all included WT and KO groups. We questioned whether GenKI is able to reveal gene functions of any target gene from a standalone WT scRNA-seq data set without pairing it with a KO counterpart, which should be a more common occurrence when using virtual KO tools. We obtained a data set from a study of 19 patients with severe coronavirus disease 2019 (COVID-19) (54). It contains 8920 cells collected from nasopharyngeal and bronchial samples. The study found that epithelial cells of COVID-19 patients showed an average three-fold increase in expression of the SARS-CoV-2 entry receptor ACE2, and signal transducer and activator of transcription 1 (STAT1), a central transcription factor of the interferon response, was among the top predictors for ACE2 expression. Previous research also shows that STAT1 is critical for virus clearance and disease resolution, and STAT1-KO mice have impaired interferon gamma (IFNG) signaling (55). In this virtual KO experiment, we focused on a subpopulation of pulmonary epithelial cells differentiating from immature secretory cells to ciliated cells. The original study demonstrated an alternative differentiation pathway leading from immature secretory cells directly into ciliated cells mediated by these IFNG-responsive epithelial cells, suggesting that this direct differentiation pathway is dependent on the interferon response (54).

We virtually knocked out STAT1 in these epithelial cells. Firstly, we validated the robustness of our model by artificially adding different levels of random noise to the gene expression profile (Supplementary Figure S8). The GenKI analysis identified 28 STAT1-KO responsive genes (Supplementary Table S10). STAT1 was ranked at the top, followed by three human leukocyte antigen (HLA) genes (HLA-DRA, HLA-DRB1, HLA-DPA1), which are known to encode Class II major histocompatibility complex (class II MHC). Class II MHC, which are reported to be highly expressed only in antigen-presenting cells (APC), is induced in other cell types as well by inflammation or IFNG (56). Moreover, lysosomes are required for lysis of the protein into peptides for class II MHC presentation to the immune cells (57). In our inferred gene list, the lysosome-related genes CTSB, CTSD, and CSTB were included, and were related to the antigen-presenting process. Previous research indicates that the nuclear factor-κB (NF-κB) can be activated by IFNG (58). This is consistent with genes in the list believed to participate in NF-κB-related pathways and inflammation. For example, ANXA1 is reported to have anti-inflammation activity in lung endothelial cells and is able to prevent lung fibrosis (59). GPX1 participates in the NF-κB pathway and is crucial for respiratory virus infection (60). S100 family proteins are well-characterized for their function in inflammation and innate immunity (61). Additionally, S100 proteins are damage-associated molecular patterns (DAMP) that promote inflammation by binding to the pattern-recognition protein (PRR) (62). HSPB1 and HSPB5 belonging to DAMP are also listed.

**Figure 7.**
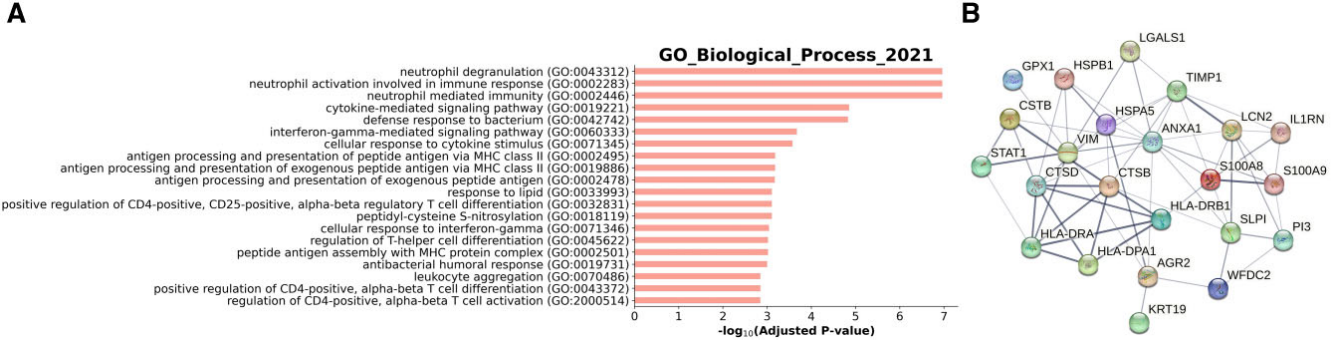
STAT1-KO responsive genes inferred by GenKI. (**A**) GO terms significantly enriched in functions of STAT1-KO responsive genes. The –log_10_-transformed adjusted *p-*value indicates the strength of enrichment for each term. (**B**) STRING network consists of STAT1-KO responsive genes.

The result of GO enrichment analysis is presented in Figure 7A and Supplementary Table S11. The neutrophil-related pathways were ranked at the very top in the enrichment analysis, suggesting the communication between IRC and neutrophils, which is in agreement with the finding of the original study (54). The interferon-gamma, class II MHC antigen-presenting, NF-κB, and innate immunity-related pathways were also detected by the enrichment analysis. Thus, these results strongly suggest that the GenKI is able to accurately predict the potential perturbed genes and their shared functions. We further analyzed 28 genes using STRING to understand their interaction (Figure 7B). The resulting subnetwork, which contains significantly more interactions than expected (*p-*value < 0.01, STRING interaction enrichment test), again suggests that these genes are closely connected due to their shared biological functions. This virtual KO study demonstrates that GenKI can reliably predict gene functions and infer the molecular phenotypic consequences of genes of interest validated by previous studies without the need for an actual KO experiment.

### GenKI is robust and scalable

To assess the robustness of GenKI inference, we collected scRNA-seq data (63) from mouse neurons with Rett syndrome (RTT), a severe neurodevelopmental disorder. Mutations in Mecp2, a transcriptional repressor required to maintain normal neuronal functions, are known to cause RTT (64,65). This data set contains two replicates with 2054 and 2156 neurons, respectively. We independently analyzed these two replicates with GenKI, in which we virtually knocked out the same KO gene Mecp2. Given the high similarity of these two biological replicates, GenKI would be robust if it generated roughly equivalent gene ranks across them. Indeed, we found high consistency between the rankings of the two reported rank lists (Spearman's correlation coefficient ρ = 0.82).

Finally, we evaluated the computation efficiency of GenKI. Supplementary Figure S9 shows the results of the analysis, comparing the total running time with respect to different sizes of input scRNA-seq data sets. The running time for GenKI consists of scGRN construction, training, and inference. We simulated four random data sets at different scales for this comparison. Without using GPUs, GenKI exhibited a 2.8- to 4.9-fold faster running speed than scTenifoldKnk tested on equivalent hardware. GenKI is expected to run even faster by enabling the fast GPU implementation optimized by PyTorch Geometric (66).

## DISCUSSION

In this study, we showcased the functionality and performance of GenKI in virtual KO experiments. We first evaluated the inference performance of GenKI using simulated data sets (SERGIO and BEELINE). Next, we used scRNA-seq data sets generated in real KO experiments to show that GenKI could predict gene functions by identifying and annotating KO-responsive genes. The functional predictions were found to be consistent with original studies in which WT and KO scRNA-seq data sets were generated.

Our main contribution in this work is to provide a neural network-based virtual KO analytical tool, which encodes the gene expression matrix to a latent space given its underlying scGRN. To the best of our knowledge, GenKI is the first virtual KO tool using a graph-based generative model to infer KO-responsive genes and their shared functions. Several computational tools have been developed for similar purposes to predict the effects of genetic perturbation using single-cell data. scGen (5) and CPA (6), both running in a supervised manner, require massive training data labeled with various perturbations to train their autoencoder-based models. CellOracle (7) can simulate gene expression in response to TFs perturbation by signal propagation through its inferred scGRN. However, this simulation is linear and does not quantify the level of perturbation at individual gene level. More importantly, it requires scATAC-seq data along with the corresponding scRNA-seq data to build the scGRN prior to making such an inference, which may limit its application. scTenifoldKnk (8) is the only virtual KO tool with the identical input requirements as GenKI. Like GenKI, scTenifoldKnk only requires WT scRNA-seq data for its prediction analysis. It employs manifold alignment (67) to project WT and virtual KO scGRNs to a joint low-dimensional space and calculate the differences between them. Given the minimalistic design, GenKI shares with scTenifoldKnk several key advantages such as being species agnostic—that is, they both work with scRNA-seq data from humans and animal models alike. By applying these tools directly to human data instead of surrogate animals, researchers may avoid pitfalls caused by overextending their conclusions from animal models to humans. Additionally, both GenKI and scTenifoldKnk allow any gene to be knocked out, regardless as to whether the KO genes are functionally vital or not. Knocking out a vital gene tends to cause fatal consequences and is, therefore, impractical to generate animal models for its KO.

GenKI outperforms scTenifoldKnk in the following aspects. First, scTenifoldKnk only utilizes the WT scGRN, while GenKI takes into account both the WT gene expression profile and scGRN. Second, the VGAE model, which consists of two message passing layers, collects information up to the second-order neighborhood of the network. In contrast, manifold alignment adopted in scTenifoldKnk only maintains the similarity of directly connected neighbors of the network, which results in different levels of inference power. In addition, GenKI shows better scalability, being able to process tens of thousands of cells within a reasonable time. Once the GenKI model is trained, the model can be reused for virtual KO of any genes in the data. While in order to do the same, scTenifoldKnk must re-solve the manifold alignment problem for each KO gene by eigen decomposition, which is considered computationally intensive and time-consuming. Last, GenKI avoids a pitfall in numerical computation in scTenifoldKnk. scTenifoldKnk performs a virtual KO experiment by removing the edges of a KO gene in the scGRN, which results in an asymmetric Laplacian matrix containing negative values. This potentially leads to eigenvectors of the Laplacian matrix with imaginary parts when solved by eigen decomposition. scTenifoldKnk practically adds 1 to all entries in obtained scGRNs to guarantee that all the entries are positive and only uses the real parts of obtained eigenvectors. GenKI’s architecture allows it to bypass this problem because it employs neural networks to solve the optimization problem, which has been shown to be numerically more stable than eigen decomposition (68).

We addressed the question that end users may often have, i.e. ‘Are KO-responsive genes more likely to be differentially expressed?’ DE analysis, followed by gene function enrichment analysis, are often used to identify the perturbed gene expression programs in order to understand the function of the KO gene. The problem is that the perturbation effect of the KO gene may propagate on the underlying network but may not direct reflected as observable and measurable changes in gene expression. GenKI, on the other hand, works on scGRNs directly to leverage unobservable network-level information—GenKI identifies perturbed genes through modelling underlying networks. Therefore, in contrast to DE analysis that can only detect perturbed genes with significant expression level changes, GenKI is likely to detect perturbed genes even there are less or no significant expression level changes. Perturbed genes without expression level changes are not uncommon. For instance, given a gene that is under control of multiple regulators, even if one of its regulators is knocked out, the remaining regulators may still be functioning to compensate and stabilize the given gene's expression. Additionally, with the default setting, GenKI produced fewer significant genes than a typical DE analysis, which may improve the interpretability of gene function. In conclusion, GenKI is not an alternative to DE analysis, but rather a complementary technique that produces more targeted results.

The limitations of GenKI are mostly inherited from it being virtual. GenKI cannot be used to predict the regulatory direction of KO-responsive genes, which is important in learning cell responses to external stimuli (69). If future refinements enable directional predictions, GenKI may improve with its potential ability to simulate the effect of overexpression. Also, GenKI, like scTenifoldKnk, currently performs a virtual KO experiment by removing all the edges of a KO gene in the WT scGRN. This action might be naïve given the complexity of a biological system. A virtual KO scGRN could be better modeled by simulating the virtual KO effect in a more probabilistic manner. Alternatively, there are many available priors involved in many different types of KO; hence a Bayesian treatment may facilitate the KO inference. GenKI is also inapplicable to bulk RNA-seq data, as genes in such data lose their variability in terms of gene expressions, which results difficulty in scGRN construction using PC regression and assigning expression values to node attributes in a graph. Recent advances in cell pseudo-temporal ordering enable us to map the underlying scGRNs throughout time (70,71) and eventually learn temporal KO effects including cell-cell communication (72) in a dynamic manner. GenKI can be improved by incorporating a dynamic inference module to investigate such effects on cell or organ development.

## Supplementary Material

gkad450_Supplemental_File

## Data Availability

The sources of data sets underlying this article can be found in Supplementary Table S1. No new data were generated in support of this research. A Python implementation of the GenKI framework is available at https://github.com/yjgeno/GenKI (permanent DOI: 10.5281/zenodo.7915654).
