# Supplementary material for: Gene knockout inference with variational graph autoencoder learning single-cell gene regulatory networks
Source: Nucleic Acids Res. 2023 May 29;51(13):6578–92. doi: 10.1093/nar/gkad450 (PMC10359630; doi:10.1093/nar/gkad450)

Supplementary Data

Table of Contents

Supplementary Tables ... 3
Supplementary Table S1. Summary of real scRNA-seq applications of GenKI analysis. ... 3
Supplementary Table S2. Trem2-KO responsive genes in microglia cells. ... 4
Supplementary Table S3. Pathway enrichment analysis results of Trem2-KO responsive genes in microglia cells (n = 10). ... 5
Supplementary Table S4. GO enrichment analysis results of Trem2-KO responsive genes in microglia cells (n = 20). ... 6
Supplementary Table S5. Nkx2-1-KO responsive genes in AT1 cells. ... 7
Supplementary Table S6. GO enrichment analysis results of Nkx2-1-KO responsive genes in AT1 cells (n = 20). ... 8
Supplementary Table S7. Hnf4a & Smad4-KO responsive genes in enterocytes. ... 9
Supplementary Table S8. GO enrichment analysis results of Hnf4a & Smad4-KO responsive genes in enterocytes (n = 20). ... 10
Supplementary Table S9. DE analyses of the Nkx2-1 KO experiment resulting from different DE methods. ... 11
Supplementary Table S10. STAT1-KO responsive genes in epithelial cells. ... 12
Supplementary Table S11. GO enrichment analysis results of STAT1-KO responsive genes in epithelial cells (n = 10). ... 13
Supplementary Figures ... 14
Supplementary Figure S1. Heatmap of Spearman correlation coefficients between scores of KL divergence given by GenKI across four different cutoffs. ... 14
Supplementary Figure S2. ROC curves of virtual KO experiments using three simulated SERGIO data sets. ... 15
Supplementary Figure S3. Comparing *p*-value given by GenKI and scTenifoldKnk to genes that are close or distance neighbors of a KO gene in the BEELINE data set ... 16
Supplementary Figure S4. Performance of GenKI in reconstructing scGRNs of microglia data set. ... 17
Supplementary Figure S5. KO responsive genes cannot be simply inferred from gene expression or edge weight with the KO gene. ... 18
Supplementary Figure S6. Comparison of absolute values of fold-change (FC=WT/KO) between randomly selected non-perturbed genes and KO-responsive genes given by GenKI. ... 19
Supplementary Figure S7. STRING networks of Trem2-KO responsive genes before and after including DE genes. ... 20

Supplementary Figure S8. Performance of GenKI in reconstructing scGRNs of COVID-19 data set..........21
Supplementary Figure S9. Total running time of GenKI and scTenifoldKnk with respect to data set sizes..........22

Supplementary Tables

Supplementary Table S1. Summary of real scRNA-seq applications of GenKI analysis.

Data set	Cell type of interest (cell number)	Target KO gene	Source	KO-responsive genes given by GenKI
Microglia	Microglia (648)	*Trem2*	GEO: GSE130627	*TREM2, CTSD, APOE, CD74, LYZ2, CD9, TYROBP, CD52, CTSZ, CTSB, ACTB, H2-AA, CYBA, NDUFA4*
Lung	AT1 cells (624)	*Nkx2-1*	GEO: GSE129628	*NKX2-1, IGFBP2, CD74, SFTPD, FOS, JUN, CYR61, DAG1, EGR1, NDNF, JUNB, NAPSA, AQP1, CTSH, NPC2, SFTA2, TIMP3, RGCC, DUSP1, IER2, SCGB1A1, CLIC5, S100A6, ANXA2, TMSB4X, CLDN3, KRT8, RTKN2, KRT19, NNAT, LGALS3, WFDC2, TUBA1A, LMO7, MAL, ANXA1, F3, SFTPC, TPPP3, KRT18, RPS2, ANO1, RNASE4, PRNP, SPOCK2, S100A11, DDC, LAMC2, ATP1B1, MFGE8, CLU, PHLDA1, IGFBP7, PTMA, MUC1, S100A10, HOPX, AKAP5, CYSTM1, FJX1, STMN1, EMB, PXDC1, CCND2, H2-AA, EPCAM, ID2, CD24A, GAS6, ACTN4, H2-AB1, PLOD2, RAB25, CLDN7, AGER, ARL6IP1, MBIP, TMEM108, H2-EB1, PAICS, PDLIM1, POPDC3*
Intestine	Enterocytes (502)	*Hnf4a* & *Smad4*	GEO: GSE112946	*HNF4A, SMAD4, REG3B, REG3G, DMBT1, REG1, CCK, GCG, GSTA1, UGT2B34, GCLM, SFXN1, LAMP2, TSC22D1*
COVID-19	Epithelial cells (108)	*STAT1*	EGAS00001004481	*STAT1, S100A8, HLA-DRA, S100A9, FTL, WFDC2, ANXA1, LCN2, CSTB, PPDPF, VIM, SLPI, HLA-DRB1, LYPD2, S100P, GPX1, CTSB, PI3, KRT19, HSPB1, FABP5, AGR2, LGALS1, IL1RN, CTSD, HLA-DPA1, HSPA5, TIMP1*
Nervous system	Neurons (2,054&2,156)	*Mecp2*	SRA: SRP135960	*MECP2, GAD2, GAD1, SYP, NDRG4, MAP1B, NAPB, GNB1, ENO2, VAMP2, RAB6B, EPHA5, TIMP2, SCD2, CNTN1, RTN1, SRCIN1, PGM2L1, MAP4, RAB3C, COX4I1, AES, ALCAM, IDS, MT-ND5, PSMB2, PRKCE, PSMA6, SLC24A3, CADM2, GRIN3A*

Supplementary Table S2. Trem2-KO responsive genes in microglia cells.

Genes are ranked according to their KL divergence between WT and virtual KO samples.

Genes	KL divergence
TREM2	1.84E5
CTSD	8.67
APOE	8.40
CD74	8.38
LYZ2	7.41
CD9	5.94
TYROBP	5.35
CD52	4.45
CTSZ	4.26
CTSB	4.11
ACTB	3.98
H2-AA	3.20
CYBA	2.79
NDUFA4	2.76
CX3CR1	2.65
TTR	2.47
COX6C	1.88
ENPP2	1.61
ADAP2OS	1.53
TIMP2	1.51

Supplementary Table S3. Pathway enrichment analysis results of Trem2-KO responsive genes in microglia cells (n = 10).

Term	Overlap	*p-value*	Adj. *p-value*	Genes
Interleukin-2 signaling pathway	7/847	1.1E-05	1.3E-03	*CX3CR1;CD52;CTSZ;TIMP2;CTSD;ACTB;CTSB*
Other semaphorin interactions	2/16	1.1E-04	4.0E-03	*TYROBP;TREM2*
Mitochondrial pathway of apoptosis	3/97	1.2E-04	4.0E-03	*CTSZ;CTSD;CTSB*
MHC class II antigen presentation	3/103	1.4E-04	4.0E-03	*CD74;CTSD;CTSB*
Lysosome	3/121	2.3E-04	5.0E-03	*CTSZ;CTSD;CTSB*
Adaptive immune system	5/606	2.7E-04	5.0E-03	*CD74;TYROBP;CYBA;CTSD;CTSB*
Alzheimer's disease	3/169	6.1E-04	9.8E-03	*NDUFA4;APOE;COX6C*
Semaphorin interactions	2/66	2.0E-03	2.7E-02	*TYROBP;TREM2*
FSH regulation of apoptosis	3/263	2.2E-03	2.7E-02	*CX3CR1;APOE;ACTB*
Immune system	5/998	2.5E-03	2.8E-04	*CD74;TYROBP;CYBA;CTSD;CTSB*

Supplementary Table S4. GO enrichment analysis results of Trem2-KO responsive genes in microglia cells (n = 20).

Term	Overlap	*p-value*	Adj. *p-value*	Genes
regulation of hippocampal neuron apoptotic process	3/5	8.5E-09	5.5E-06	*CX3CR1;TYROBP;TREM2*
macrophage activation involved in immune response	3/13	2.4E-07	3.8E-05	*CX3CR1;TYROBP;TREM2*
neutrophil degranulation	7/481	2.6E-07	3.8E-05	*TYROBP;TTR;CTSZ;TIMP2;CYBA;CTSD;CTSB*
neutrophil activation involved in immune response	7/485	2.8E-07	3.8E-05	*TYROBP;TTR;CTSZ;TIMP2;CYBA;CTSD;CTSB*
neutrophil mediated immunity	7/488	2.9E-07	3.8E-05	*TYROBP;TTR;CTSZ;TIMP2;CYBA;CTSD;CTSB*
microglial cell activation	3/22	1.3E-06	1.4E-04	*CX3CR1;TYROBP;TREM2*
positive regulation of transport	4/91	1.8E-06	1.7E-04	*CYBA;TREM2;APOE;ACTB*
regulation of interleukin-6 production	4/110	3.9E-06	3.2E-04	*CD74;TYROBP;CYBA;TREM2*
regulation of microglial cell mediated cytotoxicity	2/5	9.4E-06	6.3E-04	*CX3CR1;TYROBP*
innate immune response	5/302	9.8E-06	6.3E-04	*CX3CR1;CD74;TYROBP;CYBA;TREM2*
positive regulation of amyloid-beta clearance	2/7	1.9E-05	1.1E-03	*TREM2;APOE*
regulation of microglial cell migration	2/7	1.9E-05	1.1E-03	*CX3CR1;TREM2*
negative regulation of long-term synaptic potentiation	2/8	2.6E-05	1.3E-03	*TYROBP;APOE*
lipoprotein metabolic process	2/9	3.4E-05	1.5E-03	*APOE;CTSD*
synapse pruning	2/9	3.4E-05	1.5E-03	*CX3CR1;TREM2*
regulation of toll-like receptor 2 signaling pathway	2/11	5.2E-05	2.0E-03	*CYBA;TREM2*
lipoprotein catabolic process	2/11	5.2E-05	2.0E-03	*APOE;CTSD*
positive regulation of interleukin-6 production	3/76	5.7E-05	2.1E-03	*CD74;TYROBP;CYBA*
negative regulation of amyloid fibril formation	2/12	6.2E-05	2.1E-03	*TREM2;APOE*
negative regulation of cell activation	2/14	8.6E-05	2.8E-03	*TREM2;APOE*

Supplementary Table S5. Nkx2-1-KO responsive genes in AT1 cells.

Genes are ranked according to their KL divergence between WT and virtual KO samples.

Genes	KL divergence	Genes	KL divergence	Genes	KL divergence
NKX2-1	49.89	*LGALS3*	2.87E-05	*STMN1*	2.50E-05
IGFBP2	3.75E-05	*WFDC2*	2.86E-05	*EMB*	2.49E-05
CD74	3.73E-05	*TUBA1A*	2.84E-05	*PXDC1*	2.46E-05
SFTPD	3.65E-05	*LMO7*	2.84E-05	*CCND2*	2.46E-05
FOS	3.64E-05	*MAL*	2.81E-05	*H2-AA*	2.45E-05
JUN	3.60E-05	*ANXA1*	2.81E-05	*EPCAM*	2.44E-05
CYR61	3.35E-05	*F3*	2.80E-05	*ID2*	2.43E-05
DAG1	3.34E-05	*SFTPC*	2.79E-05	*CD24A*	2.37E-05
EGR1	3.32E-05	*TPPP3*	2.76E-05	*GAS6*	2.36E-05
NDNF	3.31E-05	*KRT18*	2.75E-05	*ACTN4*	2.35E-05
JUNB	3.26E-05	*RPS2*	2.73E-05	*H2-AB1*	2.35E-05
NAPSA	3.26E-05	*ANO1*	2.70E-05	*PLOD2*	2.33E-05
AQP1	3.25E-05	*RNASE4*	2.70E-05	*RAB25*	2.31E-05
CTSH	3.25E-05	*PRNP*	2.69E-05	*CLDN7*	2.27E-05
NPC2	3.19E-05	*SPOCK2*	2.69E-05	*AGER*	2.27E-05
SFTA2	3.13E-05	*S100A11*	2.68E-05	*ARL6IP1*	2.24E-05
TIMP3	3.12E-05	*DDC*	2.65E-05	*MBIP*	2.20E-05
RGCC	3.10E-05	*LAMC2*	2.65E-05	*TMEM108*	2.11E-05
DUSP1	3.05E-05	*ATP1B1*	2.64E-05	*H2-EB1*	2.08E-05
IER2	3.03E-05	*MFGE8*	2.64E-05	*PAICS*	2.05E-05
SCGB1A1	3.03E-05	*CLU*	2.63E-05	*PDLIM1*	2.00E-05
CLIC5	3.02E-05	*PHLDA1*	2.62E-05	*POPDC3*	1.99E-05
S100A6	2.95E-05	*PTMA*	2.61E-05		
ANXA2	2.93E-05	*IGFBP7*	2.61E-05		
TMSB4X	2.93E-05	*MUC1*	2.61E-05		
CLDN3	2.93E-05	*S100A10*	2.60E-05		
KRT8	2.91E-05	*HOPX*	2.58E-05		
RTKN2	2.90E-05	*AKAP5*	2.55E-05		
KRT19	2.90E-05	*CYSTM1*	2.52E-05		
NNAT	2.88E-05	*FJX1*	2.51E-05		

Supplementary Table S6. GO enrichment analysis results of Nkx2-1-KO responsive genes in AT1 cells (n = 20).

Term	Overlap	*p-value*	Adj. *p-value*	Genes
positive regulation of macromolecule metabolic process	12/384	5.2E-08	6.1E-05	*CD74;EGR1;CLDN3;RGCC;ANXA2;ID2;NKX2-1;MBIP;CTSH;GAS6;CLU;F3*
chemical homeostasis within a tissue	3/7	2.3E-06	7.2E-04	*NAPSA;SFTPD;CTSH*
surfactant homeostasis	3/7	2.3E-06	7.2E-04	*NAPSA;SFTPD;CTSH*
regulation of cell population proliferation	14/764	2.5E-06	7.2E-04	*JUN;RTKN2;LAMC2;CLU;CLDN3;CCND2;RGCC;RAB25;EPCAM;ID2;CTSH;IGFBP7;JUNB;S100A11*
positive regulation of cell population proliferation	11/474	3.6E-06	8.2E-04	*CD74;CCND2;RAB25;EPCAM;ID2;RTKN2;S100A6;CTSH;LAMC2;GAS6;AQP1*
positive regulation of gene expression	11/482	4.3E-06	8.2E-04	*CD74;EGR1;CLDN3;RGCC;ID2;NKX2-1;MBIP;CTSH;GAS6;CLU;F3*
positive regulation of fibroblast proliferation	4/28	4.9E-06	8.3E-04	*CD74;S100A6;GAS6;AQP1*
regulation of cell migration	10/408	6.5E-06	9.4E-04	*CD74;ANXA1;CLDN3;TMSB4X;DAG1;NKX2-1;CTSH;LAMC2;ACTN4;F3*
cellular protein metabolic process	10/417	7.8E-06	1.0E-03	*NAPSA;SFTPC;SFTPD;IGFBP2;CTSH;PLOD2;IGFBP7;RPS2;GAS6;MFGE8*
regulation of apoptotic process	13/742	9.5E-06	1.0E-03	*CD74;JUN;ANXA1;ARL6IP1;ACTN4;CLU;AQP1;KRT18;CCND2;CTSH;GAS6;PHLDA1;PTMA*
negative regulation of cytokine production	7/182	9.8E-06	1.0E-03	*PRNP;ANXA1;RGCC;TMSB4X;SFTPD;GAS6;AGER*
negative regulation of programmed cell death	9/381	2.6E-05	2.5E-03	*CD74;ANXA1;KRT18;CCND2;ARL6IP1;CTSH;GAS6;PTMA;AQP1*
negative regulation of apoptotic process	10/485	2.9E-05	2.6E-03	*CD74;ANXA1;KRT18;CCND2;ARL6IP1;CTSH;NDNF;GAS6;PTMA;AQP1*
regulation of fibroblast proliferation	4/46	3.8E-05	3.1E-03	*CD74;S100A6;GAS6;AQP1*
positive regulation of neuron death	4/47	4.1E-05	3.2E-03	*EGR1;PRNP;FOS;CLU*
positive regulation of cell migration	7/269	1.2E-04	7.9E-03	*CLDN3;RAB25;CTSH;LAMC2;ACTN4;F3;S100A11*
cellular response to reactive oxygen species	4/63	1.3E-04	7.9E-03	*JUN;ANXA1;FOS;AQP1*
positive regulation of cell death	4/66	1.5E-04	8.0E-03	*EGR1;PRNP;FOS;CLU*
negative regulation of intrinsic apoptotic signaling pathway in response to DNA damage	3/26	1.6E-04	8.0E-03	*CD74;MUC1;CLU*
positive regulation of G1/S transition of mitotic cell cycle	3/26	1.6E-04	8.0E-03	*CCND2;ANXA1;RGCC*

Supplementary Table S7. Hnf4a & Smad4-KO responsive genes in enterocytes.

Genes are ranked according to their KL divergence between WT and virtual KO samples.

Genes	KL divergence
HNF4A	7.03E2
SMAD4	10.57
REG3B	1.29E-02
REG3G	1.09E-02
DMBT1	4.18E-03
REG1	3.05E-03
CCK	1.91E-03
GCG	1.43E-03
GSTA1	1.05E-03
UGT2B34	3.34E-04
GCLM	3.33E-04
SFXN1	3.02E-04
LAMP2	2.90E-04
TSC22D1	2.58E-04

Supplementary Table S8. GO enrichment analysis results of Hnf4a & Smad4-KO responsive genes in enterocytes (n = 20).

Term	Overlap	*p-value*	Adj. *p-value*	Genes
glutathione metabolic process	2/43	4.0E-04	3.8E-02	*GSTA1;GCLM*
defense response to Gram-positive bacterium	2/70	1.0E-03	3.8E-02	*DMBT1;REG3G*
carbohydrate homeostasis	2/70	1.1E-03	3.8E-02	*HNF4A;GCG*
regulation of peptide hormone secretion	2/74	1.2E-03	3.8E-02	*HNF4A;GCG*
glucose homeostasis	2/86	1.6E-03	3.8E-02	*HNF4A;GCG*
epithelial cell differentiation	2/101	2.2E-03	3.8E-02	*DMBT1;GSTA1*
regulation of insulin secretion	2/104	2.3E-03	3.8E-02	*HNF4A;GCG*
sulfur compound biosynthetic process	2/113	2.8E-03	3.8E-02	*GSTA1;GCLM*
epithelium development	2/122	3.2E-03	3.8E-02	*DMBT1;GSTA1*
regulation of protein secretion	2/125	3.3E-03	3.8E-02	*HNF4A;GCG*
negative regulation of cell growth	2/126	3.4E-03	3.8E-02	*SMAD4;HNF4A*
negative regulation of growth	2/126	3.4E-03	3.8E-02	*SMAD4;HNF4A*
positive regulation of cell proliferation involved in heart morphogenesis	1/5	3.5E-03	3.8E-02	*SMAD4*
lysosomal protein catabolic process	1/5	3.5E-03	3.8E-02	*LAMP2*
negative regulation of keratinocyte differentiation	1/5	3.5E-03	3.8E-02	*REG3G*
positive regulation of histone H3-K9 acetylation	1/5	3.5E-03	3.8E-02	*SMAD4*
cardiac endothelial cell differentiation	1/5	3.5E-03	3.8E-02	*SMAD4*
endocardial cell differentiation	1/5	3.5E-03	3.8E-02	*SMAD4*
negative regulation of cardiac muscle cell differentiation	1/6	4.2E-03	3.8E-02	*SMAD4*
induction of bacterial agglutination	1/6	4.2E-03	3.8E-02	*DMBT1*

Supplementary Table S9. DE analyses of the Nkx2-1 KO experiment resulting from different DE methods.

DE method	Wilcoxon	Wilcoxon (logFC>1)	t-test	negbinom	MAST	DESeq2
Input data	Normalized data	Normalized data	Normalized data	Raw counts	Normalized data	Raw counts
Model	Nonparametric test	Nonparametric test	Parametric test	Negative binomial model	Generalized linear model	Negative binomial model
DE genes	1129	528	1019	878	1019	1137
Upregulated	273	175	293	167	293	240
Downregulated	856	353	726	711	726	897
DE intersect with KO-responsive genes	71 (17*)	49 (15)	70 (7)	69 (24)	74 (21)	81 (11)
Upregulated DE intersect	42	29	41	28	44	35
Downregulated DE intersect	29	20	29	41	30	46

*Number in bracket: intersection between KO-responsive genes and top 50 DE genes ranked by each DE method

Supplementary Table S10. STAT1-KO responsive genes in epithelial cells.

Genes are ranked according to their KL divergence between WT and virtual KO samples.

Genes	KL divergence
STAT1	2.94E3
S100A8	8.72
HLA-DRA	4.29
S100A9	2.90
FTL	1.98
WFDC2	1.59
ANXA1	1.56
LCN2	1.33
CSTB	1.21
PPDPF	1.13
VIM	1.04
SLPI	0.87
HLA-DRB1	0.83
LYPD2	0.81
S100P	0.80
GPX1	0.66
CTSB	0.61
PI3	0.59
KRT19	0.55
HSPB1	0.53
FABP5	0.52
AGR2	0.41
LGALS1	0.37
IL1RN	0.35
CTSD	0.34
HLA-DPA1	0.31
HSPA5	0.27
TIMP1	0.26

Supplementary Table S11. GO enrichment analysis results of STAT1-KO responsive genes in epithelial cells (n = 10).

Term	Overlap	*p-value*	Adj. *p-value*	Genes
neutrophil degranulation	10/481	5.2E-10	1.1E-07	*CSTB;SLPI;FABP5;LCN2;S100P;CTSD;S100A9;S100A8;CTSB;FTL*
neutrophil activation involved in immune response	10/485	5.7E-10	1.1E-07	*CSTB;SLPI;FABP5;LCN2;S100P;CTSD;S100A9;S100A8;CTSB;FTL*
neutrophil mediated immunity	10/488	6.0E-10	1.1E-07	*CSTB;SLPI;FABP5;LCN2;S100P;CTSD;S100A9;S100A8;CTSB;FTL*
cytokine-mediated signaling pathway	9/621	1.0E-07	1.4E-05	*IL1RN;ANXA1;STAT1;LCN2;HLA-DRA;TIMP1;VIM;HLA-DRB1;HLA-DPA1*
defense response to bacterium	6/176	1.4E-07	1.5E-05	*SLPI;LCN2;PI3;S100A9;WFDC2;S100A8*
interferon-gamma-mediated signaling pathway	4/68	2.4E-06	2.1E-04	*STAT1;HLA-DRA;HLA-DRB1;HLA-DPA1*
cellular response to cytokine stimulus	7/482	3.5E-06	2.7E-04	*IL1RN;ANXA1;STAT1;LCN2;TIMP1;VIM;HLA-DPA1*
antigen processing and presentation of exogenous peptide antigen via MHC class II	4/98	1.0E-05	6.6E-04	*HLA-DRA;CTSD;HLA-DRB1;HLA-DPA1*
antigen processing and presentation of peptide antigen via MHC class II	4/100	1.1E-05	6.6E-04	*HLA-DRA;CTSD;HLA-DRB1;HLA-DPA1*
response to lipid	4/114	1.8E-05	7.8E-04	*ANXA1;SLPI;S100A9;S100A8*

Supplementary Figures

Supplementary Figure S1. Heatmap of Spearman correlation coefficients between scores of KL divergence given by GenKI across four different cutoffs.

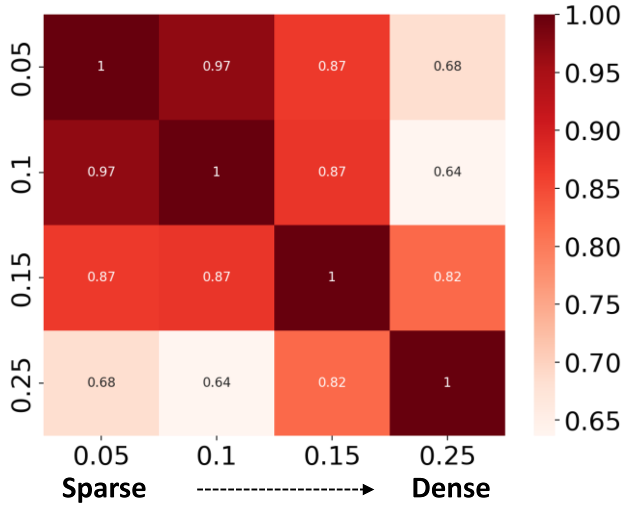

Supplementary Figure S2. ROC curves of virtual KO experiments using three simulated SERGIO data sets.

(**A**) KO gene #74 (35 edges) in SERGIO simulated data set of 100 genes by 2,700 cells. (**B**) KO gene #47 (110 edges) in SERGIO simulated data set of 400 genes by 2,700 cells. (**C**) KO gene #302 (135 edges) in SERGIO simulated data set of 1,200 genes by 2,700 cells.

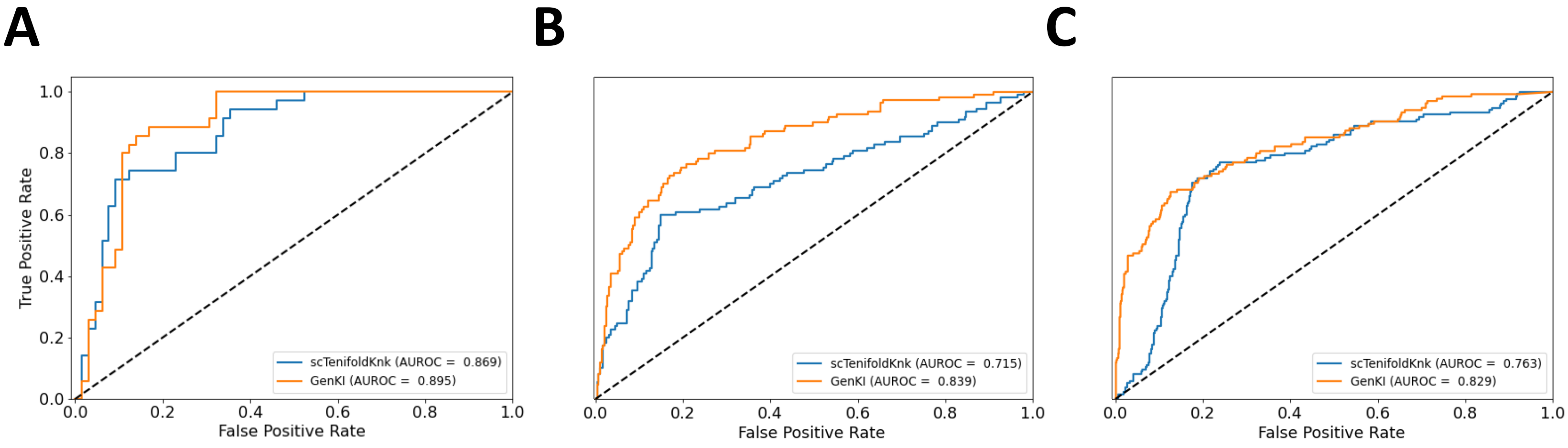

Supplementary Figure S3. Comparing *p*-value given by GenKI and scTenifoldKnk to genes that are close or distance neighbors of a KO gene in the BEELINE data set.

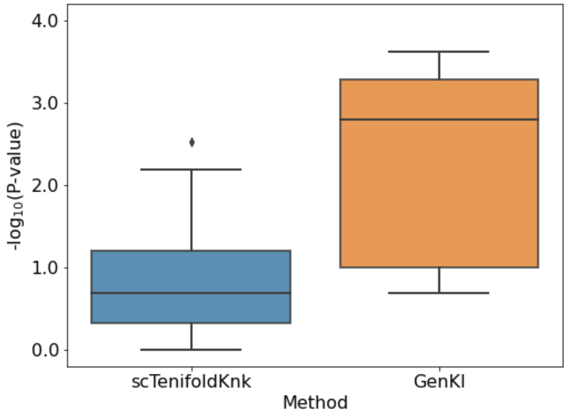

Supplementary Figure S4. Performance of GenKI in reconstructing scGRNs of microglia data set.

(**A**) The levels of AP at different training epochs. (**B**) The APs between data with different levels of random noise in the gene expression profiles. Larger σ values result in higher levels of random noise (see "**Materials and Methods**" for details). (**C**) The levels of AUROC at different training epochs. (**D**) The AUROCs between data with different levels of random noise in the gene expression profiles.

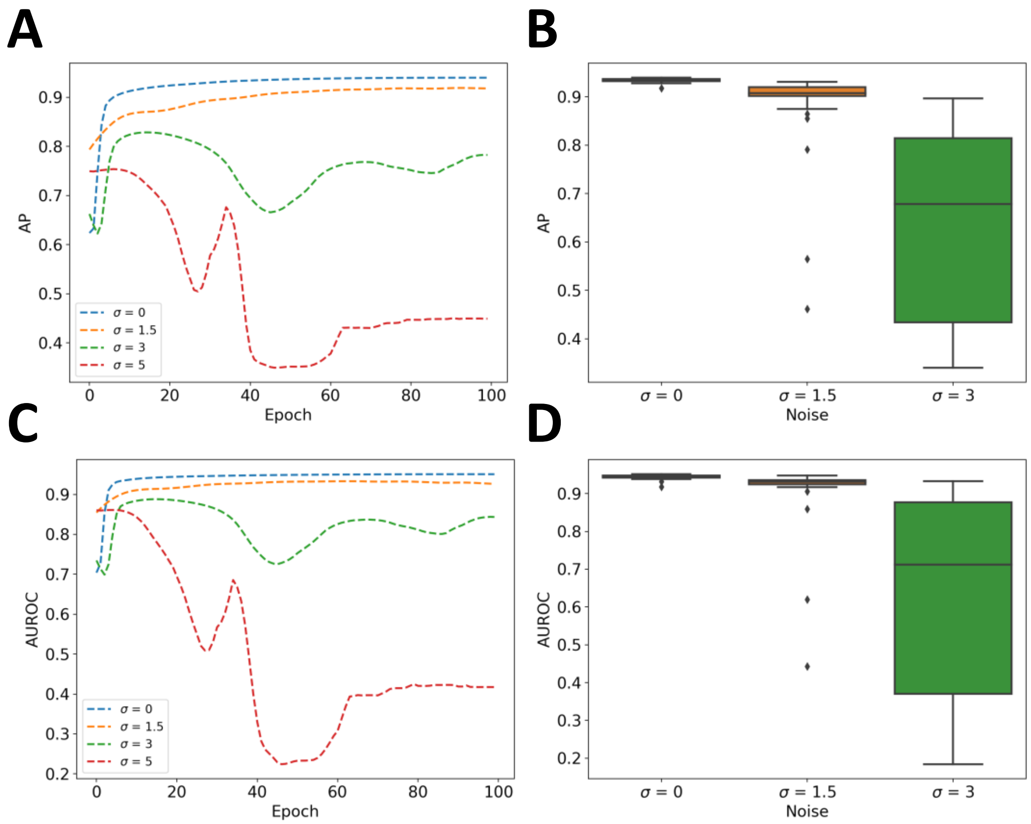

Supplementary Figure S5. KO responsive genes cannot be simply inferred from gene expression or edge weight with the KO gene.

(**A**) Scatter plot revealing the relationship between normalized gene expression and KL divergence. (**B**) Scatter plot revealing the relationship between edge weight associated with Trem2 and KL divergence. The KO-responsive genes inferred by GenKI are annotated and Trem2 as an outlier is ignored for visualization purpose.

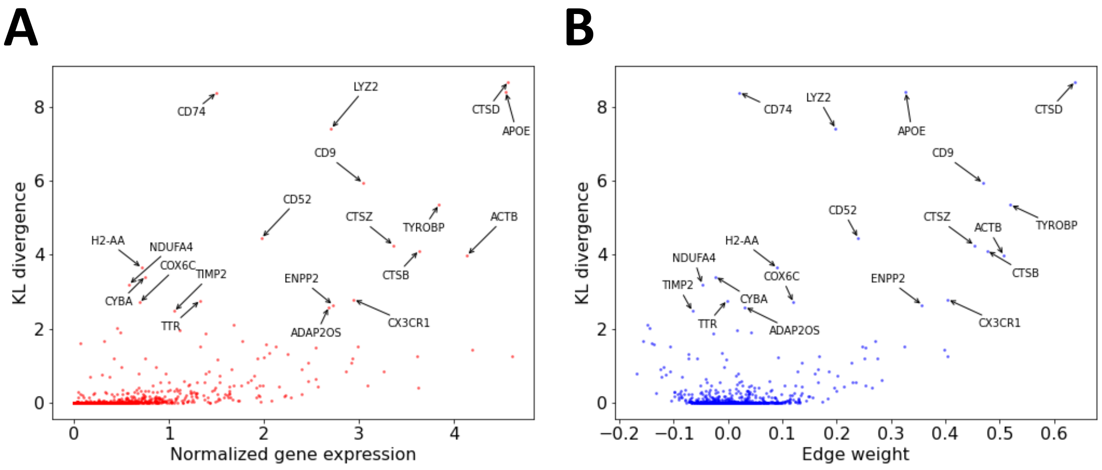

Supplementary Figure S6. Comparison of absolute values of fold-change (FC=WT/KO) between randomly selected non-perturbed genes and KO-responsive genes given by GenKI.

(**A**) Microglia data set (**B**) Lung data set (**C**) Intestine data set. *P*-values of the one-sided t-test are 0.0168, 0.0177 and 0.0175, respectively.

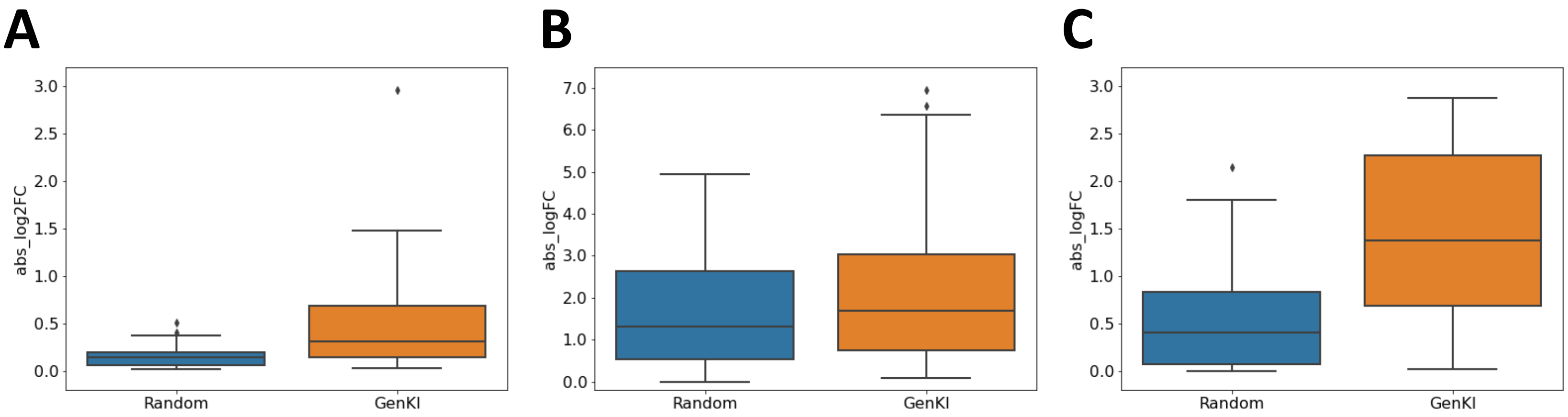

Supplementary Figure S7. STRING networks of Trem2-KO responsive genes before and after including DE genes.
(**A**) Trem2-KO responsive genes (in green) without DE genes (**B**) Trem2-KO responsive genes with top ranked DE genes (in red).

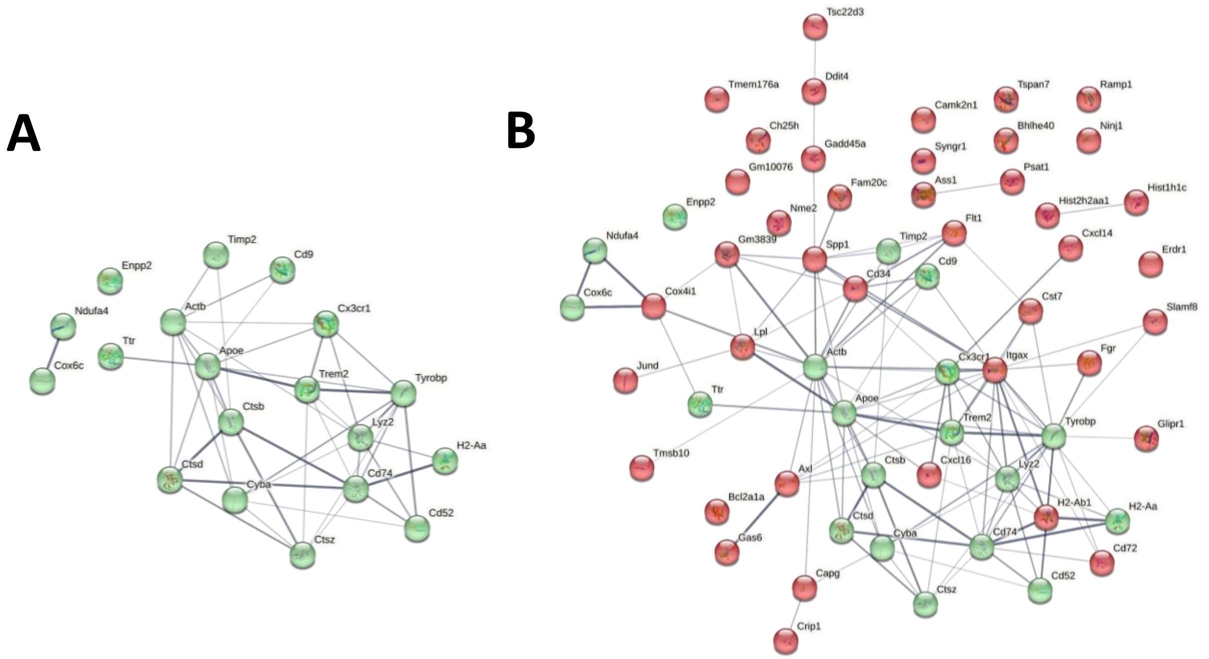

Supplementary Figure S8. Performance of GenKI in reconstructing scGRNs of COVID-19 data set.
(**A**) The levels of AP at different training epochs. (**B**) The levels of AUROC at different training epochs. Larger σ values result in higher levels of random noise (see "**Materials and Methods**" for details).

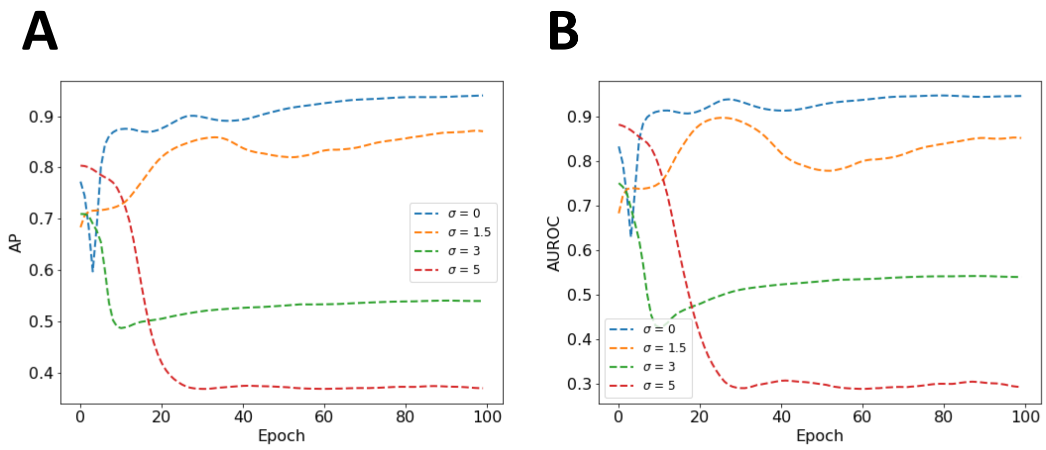

Supplementary Figure S9. Total running time of GenKI and scTenifoldKnk with respect to data set sizes.

All evaluations were implemented on the equivalent hardware composed of a 16-core Intel Core i7-11700 CPU processor at 2.50 GHz with 15.7 GB available random-access memory (RAM). PyTorch profiler was used for GenKI and the system.time function for scTenifoldKnk.

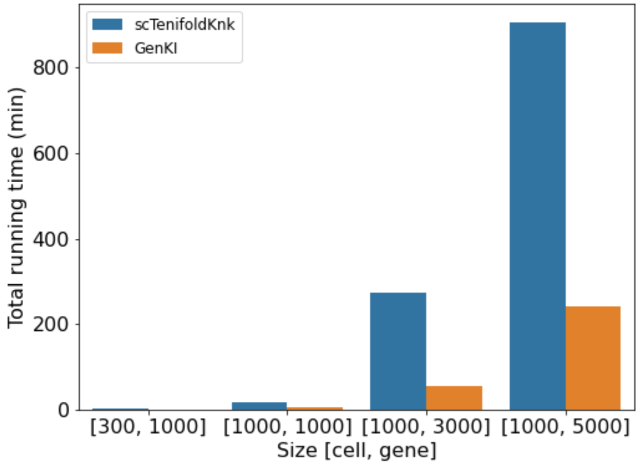

Supplement: gkad450_Supplemental_File [file gkad450_supplemental_file.pdf]
